# A shift in the cellular redox state redirects aspartate for export under glucose deprivation

**DOI:** 10.1186/s40170-026-00420-x

**Published:** 2026-03-03

**Authors:** Barbara Konrad, Gabriele Bluemel, Theresa Haitzmann, Tobias Frech, Anke Vandekeere, Mélanie Planque, Visnja Bubalo, Katharina Schindlmaier, Vanessa Jäger, Michael A. Dengler, Sarah Stryeck, Luka Brcic, Jörg Lindenmann, Philipp Stiegler, Doruntina Bresilla, Corina T. Madreiter-Sokolowski, Tobias Madl, Thomas O. Eichmann, Nikolaus Kneidinger, Sarah-Maria Fendt, Katharina Leithner

**Affiliations:** 1 Division of Respiratory Medicine, Department of Internal Medicine, Medical University of Graz, Auenbruggerplatz 15, Graz, 8036 Austria; 2 Department of Biosciences and Medical Biology, Bioanalytical Research Labs, University of Salzburg, Hellbrunner Strasse 34, Salzburg, 5020 Austria; 3 Division of Pharmacology, Otto Loewi Research Center, Medical University of Graz, Neue Stiftingtalstraße 6, Graz, 8010 Austria; 4 Laboratory of Cellular Metabolism and Metabolic Regulation, VIB-KU Leuven Center for Cancer Biology, VIB, Herestraat 49, Leuven, 3000 Belgium; 5 Laboratory of Cellular Metabolism and Metabolic Regulation, Department of Oncology, KU Leuven and Leuven Cancer Institute (LKI), Herestraat 49, Leuven, 3000 Belgium; 6 Division of Oncology, Department of Internal Medicine, Medical University of Graz, Auenbruggerplatz 15, Graz, 8036 Austria; 7 Division of Molecular Biology and Biochemistry, Gottfried Schatz Research Center, Medical University of Graz, Neue Stiftingtalstraße 6, Graz, 8010 Austria; 8 Diagnostic and Research Institute of Pathology, Medical University of Graz, Neue Stiftingtalstraße 6, Graz, 8010 Austria; 9 Division of Thoracic and Hyperbaric Surgery, Department of Surgery, Medical University of Graz, Auenbruggerplatz 29/3, Graz, 8036 Austria; 10 Lung Research Cluster, Medical University of Graz, Neue Stiftingtalstraße 6, Graz, 8010 Austria; 11 Division of General, Visceral and Transplant Surgery, Department of Surgery, Medical University of Graz, Auenbruggerplatz 5, Graz, 8036 Austria; 12 Division of Medicinal Chemistry, Otto Loewi Research Center, Medical University of Graz, Neue Stiftingtalstraße 6, Graz, 8010 Austria; 13 BioTechMed-Graz, Mozartgasse 12/II, Graz, 8010 Austria; 14 Core Facility Mass Spectrometry, Medical University of Graz, Neue Stiftingtalstraße 6, Graz, 8010 Austria; 15 Present Address: Department of Pathology, Hospital Graz II, Graz, Austria; 16 Present Address: Department of Pathology, Medical University of Vienna, Vienna, Austria

**Keywords:** Cancer, Metabolism, Adaptation, Glucose deprivation, Aspartate, Aspartic acid

## Abstract

**Background:**

Glucose is an important fuel in cancer cells, however, its availability may be limited in solid tumors. Cell-autonomous, metabolic adaptations of cancer cells and non-malignant cells to glucose deprivation are still incompletely understood.

**Methods:**

Here, we addressed the changes in central carbon metabolism in lung cancer cells and normal lung cells facing glucose limitation using stable isotopic labeling followed by nuclear magnetic resonance spectroscopy and mass spectrometry.

**Results:**

Elevated levels and the release of newly synthesized aspartate were among the most prominent changes in low compared to high glucose conditions. The low glucose-induced export of aspartate occurred in different lung cancer cell lines, but also bronchial epithelial cells and cancer-associated fibroblasts. It was accompanied by a reduced use of aspartate in purine synthesis and suppressed by hypoxia. A knockout of the malate-aspartate shuttle (MAS) enzyme mitochondrial aspartate aminotransferase (GOT2) decreased aspartate release. Low glucose conditions diminished reduced nicotinamide adenine dinucleotide (NADH) and restoring NADH reversed aspartate synthesis, suggesting that the distal, NADH-dependent arm of the MAS is compromised under glucose deprivation.

**Conclusions:**

Cells accumulate and release aspartate, a biosynthetic precursor and signaling molecule, under low glucose conditions, largely due to a truncated MAS, as part of their adaptive metabolic response.

**Supplementary Information:**

The online version contains supplementary material available at 10.1186/s40170-026-00420-x.

## Introduction

Glucose is a precursor for important biosynthetic pathways in rapidly proliferating cancer and immune cells [1–3]. However, glucose levels are typically found to be reduced in the tumor microenvironment compared to plasma levels [4–7], and steep gradients may occur, partly due to a poor vascular supply [8, 9]. To understand the relationship between nutrient availability and cell metabolism is key to exploit metabolic dependencies of cancer cells as targets for therapy [10]. In microbes, central carbon metabolic fluxes are self-adjusted to nutritional changes [11], however, the cell-autonomous changes in malignant cells and accompanying normal cells to glucose deprivation are still incompletely understood.

Glycolytic intermediates are diverted to produce crucial cellular building blocks, like ribose-phosphate for DNA synthesis, glycerol-phosphate for glycerolipid synthesis or serine and glycine, to generate one-carbon units. Pyruvate produced from glycolysis is then either converted to lactate or to acetyl-CoA to fuel the tricarboxylic acid (TCA) [1–3]. Besides donating reducing equivalents to the respiratory chain, the TCA cycle generates important anabolic precursors, especially aspartate [1–3]. Aspartate is required for the synthesis of nucleotides [12, 13] and indeed, one of the functions of mitochondrial respiration in cancer cells is to allow aspartate synthesis [14, 15]. However, the synthesis of aspartate is also linked to an important redox shuttle, the malate-aspartate shuttle (MAS). The MAS canonically starts with the conversion of the TCA cycle intermediate oxaloacetate (OAA) to aspartate by the mitochondrial isoform of aspartate aminotransferase (GOT2). Aspartate is then exported to the cytoplasm and further converted back to OAA by cytoplasmic GOT1, followed by the reduction of OAA to malate by cytoplasmic malate dehydrogenase (MDH1). The latter requires reduced nicotinamide adenine dinucleotide (NADH). Malate is then transferred back into the mitochondria and re-oxidized to OAA by MDH2, thus re-generating NADH and OAA in the mitochondria. The MAS transports reducing equivalents from cytosolic NADH to the mitochondrial electron transport chain [16], since the mitochondrial inner membrane is impermeable to NADH [17]. The shuttle is especially important in glycolytic cells to ensure the regeneration of NAD^+^ from NADH formed in the glyceraldehyde-3-phosphate dehydrogenase (GAPDH) step in glycolysis [16]. How the interplay between glycolysis and the MAS is changed upon acute glucose deprivation is still unclear.

Despite the need for aspartate, cancer cells have been shown to release aspartate [18] and aspartate has been found to be elevated in the tumor microenvironment of genetically engineered pancreatic adenocarcinoma (PDAC) in mice compared to plasma [5]. Recently, treatment of cancer cells with both, an inhibitor targeting class I glucose transporters (GLUTs) or complete removal of glucose, was shown to dramatically enhance the cellular levels of aspartate, along with a shift of the NAD^+^/NADH ratio towards NAD^+^ [19]. Addressing metabolic adaptations in lung cancer cells, normal lung cells and cancer-associated fibroblasts, we found that the accumulation and release of *de novo* synthesized aspartate are among the most prominent changes in cells facing a low availability of glucose. Moreover, we show that a truncated MAS caused by a decline in NADH is largely responsible for the release of aspartate.

## Materials and methods

### Cancer cell lines

The human lung cancer cell line A549 was obtained from Cell Lines Service (Eppelheim, Germany). A549 cells were cultured in DMEM/F-12 (Gibco, Thermo Fisher Scientific, Waltham, MA, USA) supplemented with 2 mM glutamine (Gibco), 10% fetal bovine serum (FBS, Biowest, Nuaillé, France) and antibiotics (Gibco). The human lung cancer cell line NCI-H23 was purchased from the American Type Culture Collection (ATCC, Manassas, VA, USA). NCI-H460 cells were a kind gift from Martin P. Barr, Institute of Molecular Medicine, St. James’s Hospital and Trinity College Dublin, Dublin, Ireland. H23 cells were cultured in RPMI 1640 (Gibco) supplemented with 2 mM glutamine, 10% FBS and antibiotics. H460 cells were cultured in DMEM containing 10 mM glucose and 2 mM glutamine (Gibco) supplemented with 10% fetal calf serum (Biowest) and antibiotics. Cell line authentication was done for all cell lines by Short Tandem Repeat (STR) analysis using the PowerPlex 16HS System (Promega, Madison, WI, USA).

### Cancer-associated fibroblasts

Cancer-associated fibroblasts (CAFs) were isolated from three fresh non-small cell lung cancer samples obtained from surgery as described [20] and cultured in DMEM containing 5 mM glucose and 2 mM glutamine (Gibco) supplemented with 10% fetal calf serum (Biowest) and antibiotics. The isolation of CAFs has been conducted according to the Declaration of Helsinki principles and was approved by the institutional Ethics Committee. Signed informed consent was obtained from all patients prior to surgery.

### Bronchial epithelial cells

Primary bronchial epithelial cells (BEC) were isolated from non-utilized human donor lungs, explanted at the Department of Surgery, Medical University of Graz, Austria (P.S.) according to a protocol modified after Fulcher et al. [21]. The isolation of BECs has been conducted according to the Declaration of Helsinki principles and was approved by the institutional Ethics Committee. Briefly, proximal to segmental bronchi from explant lungs were cut in approx. 1 × 2 cm pieces, rinsed with PBS and the epithelium was gently scraped. The cells were resuspended in DMEM/F12 medium supplemented with 20% FBS, antibiotics and antimycotic (Fungizone, 1:100; Gibco) and the suspension was transferred onto a cell culture dish coated with collagen type I (rat-tail collagen, 3 mg/mL, Gibco, diluted 1:100 in PBS). After cell attachment, the medium was replaced with bronchial epithelium growth medium (BEGM, CC-3170, Lonza, Basel, Switzerland) containing growth factors. Cells were grown under standard conditions at 37 °C, 5% CO_2_ and 95% humidity and used in low passages (≤5). BEC were 99.9% cytokeratin positive upon staining with a pan-cytokeratin, mouse monoclonal antibody (Clone: MNF116, 1:100, Invitrogen, Thermo Fisher Scientific).

### Glucose deprivation and hypoxia experiments

For glucose deprivation experiments, cells were plated in growth medium. After 24 hours, cells were washed two times with PBS and treated with high or low glucose media. In H23 cells and BEC SILAC RPMI 1640 medium (Gibco) lacking glucose and glutamine was supplemented with 10 mM or 0.2 mM glucose, 2 mM glutamine, as well as arginine and lysine (Sigma-Aldrich, St. Louis, MO, USA) at concentrations present in normal RPMI 1640. BEC were additionally treated with 10% dialyzed FBS (Gibco). In all other cell types, DMEM lacking glucose and glutamine (Gibco) was used, supplemented with 2 mM glutamine and 10 mM or 0.2 mM glucose (Sigma-Aldrich). All treatment media were devoid of pyruvate except in the lactate/pyruvate experiments. dFCS was used only where indicated. For culture under hypoxia, the automated Xvivo System G300CL (BioSpherix, Lacona, NY) was set to 0.5% oxygen. Media were pre-equilibrated under hypoxia overnight and medium changes were done in a 0.5% oxygen atmosphere in the hypoxic workbench (Xvivo System G300CL, BioSpherix). In all experiments, the medium was replaced daily.

### GOT1 silencing with siRNA

Cells were transfected either with non-silencing (ctrl) siRNA (non-targeting pool, Dharmacon, Horizon, Colorado, USA) or with two different, commercially available pools of GOT1 siRNA (Dharmacon), GOT1_si1 and GOT1_si2, using jetPRIME (Polyplus, Illkirch, France) transfection reagent at a final concentration of 40 nM.

### CRISPR/Cas9 mediated gene editing

H23 and A549 GOT2 knockouts were generated by the inducible CRISPR/Cas9 gene editing system described by Aubrey et al. [22]. For a constitutive expression of the Cas9 protein, H23 and A549 cells were transduced with a pFUCas9mCherry vector, and additionally with the pFgh1tUTG lentiviral vector (doxycycline-inducible) carrying sgRNA targeting LacZ (sgLacZ, as control) or GOT2 (sgGOT2#1 or sgGOT2#2 for GOT2 knockout cells). Cells were selected using fluorescence activated cell sorting (BD FACS Aria-IIIu, BD Biosciences, Franklin Lakes, NJ). Transfection efficiency of H23 and A549 cells was determined by analysis of the fluorescent proteins GFP and mCherry and GOT2 knockout was induced by doxycycline (1 µg/mL). Western Blot analysis was performed to determine the efficacy of GOT2 knockout in the polyclonal cell lines after doxycycline-induction.

### Nuclear magnetic resonance (NMR) spectroscopy - sample preparation, data acquisition and analysis

Cell samples were stored at −80^◦^C prior analysis. To extract metabolites, 400 µL of ice-cold methanol and 200 µL MilliQ H_2_O were added, and the samples were transferred to a tube containing Precellys beads (1.4 mm zirconium oxide beads, Bertin Technologies, Villeurbanne, France) for homogenization by Precellys24 tissue homogenizer (Bertin Technologies). Samples were centrifuged at 13,000 rpm at 4^◦^C for 30 min. After centrifugation, supernatants were lyophilized at < 1 Torr, 850 rpm, 25^◦^C for 10 h in a vacuum-drying chamber (Savant Speedvac SPD210 vacuum concentrator).

For the NMR experiments, samples were re-dissolved in 500 µL of NMR buffer (0.08 M Na_2_HPO_4_, 5 mM TSP (3-(trimethylsilyl) propionic acid-2,2,3,3-d_4_ sodium salt), 0.04 (w/v)% NaN_3_ in D_2_O, pH adjusted to 7.4 with 8 M HCl and 5 M NaOH) and transferred to 5 mm NMR tubes. The metabolic-profiling analysis was performed at 310 K using a 600 MHz Bruker Avance Neo NMR spectrometer equipped with a TXI 600S3 probe head. The Carr–Purcell–Meiboom–Gill (CPMG) pulse sequence was used to acquire ^1^H 1D NMR spectra with a pre-saturation for water suppression (cpmgpr1d, 512 scans, 73728 points in F1, 12019.230 Hz spectral width, recycle delay 4s) [23, 24]. NMR spectral data were processed as previously described [25]. Shortly, data were processed in Bruker Topspin version 4.0.2 using one-dimensional exponential window multiplication of the FID, Fourier transformation and phase correction. Then, the NMR data were imported into Matlab2014b; TSP was used as the internal standard for chemical-shift referencing (set to 0 ppm); regions around the water, TSP and methanol signals were excluded; the NMR spectra were aligned; and a probabilistic quotient normalization was performed. To identify changes in metabolomic profiles, PCA, orthogonal partial least squares discriminant analysis (O-PLS-DA) and partial least squares-discriminant analysis (PLS-DA) were performed in Matlab2014b and MetaboAnalyst 4.0 [26], as well as all associated data consistency checks and cross-validation. Validation of the statistical significance of the determined differences were done by the quality assessment statistic Q^2^, which provides information about cross-validation. It is a qualitative measure of consistency between the predicted and original data with a maximum value of 1. Chenomx NMR Suite 8.4 (Chenomx Inc., Edmonton, AB, Canada) and reference compounds were used for metabolite identification. Quantification of metabolites was carried out by signal integration of normalized spectra. For each metabolite, a representative peak with no overlapping signals was identified, the start and end points of the integration were chosen to revolve around that peak, and the area of the peak was integrated by summing up the value for each point.

For analysis of the ^13^C-labeled metabolites, ^1^H,^13^C HSQC NMR experiments were recorded (pulseprogram hsqcetgpsisp2, 8 scans, 10026.738 Hz sweep width in F2, 2048 increments in F2, 12658.228 Hz sweep width in F1, 256 increments in F1). The cross peaks corresponding to the presented metabolites (Asp Hβ-Cβ) were assigned and integrated for further analyses.

### Stable isotopic tracing and gas chromatography - mass spectrometry (GC-MS)

Stable isotopic tracing, metabolite extraction and GC-MS were essentially performed as described [27, 28]. After plating and a starvation pretreatment of 24 hours, cells were treated again for further 24 hours with the respective treatment media including 2 mM ^13^C_5_-L-glutamine (Sigma Aldrich), 2 mM α−^15^N-L-glutamine (Cambridge Isotope Laboratories, Tewksbury, MA), 150 µM ^13^C_4_- L-aspartic acid (Cambridge Isotope Laboratories) or 150 µM ^15^N-L-aspartic acid (Cambridge Isotope Laboratories) as tracer. Cells were washed with 0.9% NaCl (Fresenius, Bad Homburg, DE) and quenching was performed by flash-freezing the plates on liquid nitrogen. Cells and media supernatants were extracted on ice with ice-cold 62.5% methanol in H_2_O containing norvaline or glutarate (Sigma-Aldrich) as internal standard. Ice-cold chloroform was added for the extraction and samples were sonicated (3x5 s on ice) or vortexed thoroughly. For phase-separation, samples were centrifuged (13,000 rpm, 10 min, 4°C) and the upper phase containing the polar metabolites was evaporated by vacuum centrifugation. Dried polar samples were derivatized with 20 µL methoxyamine (MOX, 20 mg/mL) reagent (Thermo Fisher Scientific or Sigma) in pyridine for 60/90 minutes at 37 °C and with additional 30/40 µL of *N*-(tert-butyldimethylsilyl)-N-methyl-trifluoroacetamide with 1% tert-butyldimethylsilyl ethers (MTBSTFA + 1% TBDMS, Thermo Fisher Scientific) for 30/60 minutes at 60 °C. Samples were analyzed using the 7890B GC system coupled to a 5977A Inert MS system (Agilent Technologies, Santa Clara, CA) or the 8860 GC system combined with an 5977C Inert MS system (Agilent Technologies). A DB-35 ms column and helium as carrier gas with a flow rate of 1 mL/min were used for chromatographic separation. The injection volume was 1 μL and measurements were taken in split-less mode. The inlet temperature was set to 270 °C. The GC oven temperature program used for the separation was as follows: the oven was held at 100 °C for 3 min, then ramped at 3.5 °C min^−1^ to 300 °C. Alternatively, the GC oven was kept at 100 °C for 1 min, increased up to 105 °C with a gradient of 2.5 °C min^−1^, and kept for 2 min, then ramped to 240 °C with a gradient of 3.5 °C min^−1^, and after that ramped up to 320 °C for 2 min with a gradient of 22 °C min^−1^ and kept for 2 min, followed by 4 min at 320 °C. For this protocol, the temperatures of the quadrupole and the source were set at 150 °C and 230°C, respectively. Mass spectra were obtained at 70 eV at a mass range of 100–650 atomic mass units. El-Maven software [29] was used for peak quantification and correction for natural abundance was performed with Isocor [30] or with a MATLAB Script (MA, USA). Calculated metabolite abundances were normalized to norvaline or glutarate and the total protein amount. For absolute quantification of aspartate in media supernatants, each unlabeled sample was spiked with ^13^C_4_-aspartate. Linearity was confirmed by standard dilutions of ^13^C_4_-aspartate.

### Liquid chromatography–mass spectrometry (LC–MS)

1.2–1.5 Mio cells were plated per 10 cm culture dish and treated as described above. Thereafter, cells were washed with 0.9% NaCl, and dishes were flash frozen. Then cells were scraped with 80% methanol including internal standards on ice (for the ^15^N experiments) and the samples were dried (SpeedVac, Thermo Fisher Scientific), resuspended and sonicated in 80% methanol (MeOH) containing internal standards if not added prior to scraping (IS, 670 pmol glutarate, Sigma-Aldrich in ^13^C labeled samples and 8 nmol 2,3-^13^C_2_-serine, Sigma-Aldrich, in ^15^N-labeled samples for purine synthesis pathway analysis) using a Bioruptor Pico (30 min, 4 °C, 30 sec ON/30 sec OFF, frequency high; Diagenode). Samples were supplemented with ddH_2_O and methyl tert-butyl ether (MTBE) and incubated under shaking for 15 minutes at 4 °C. Samples were centrifuged (13,000 rpm, 10 min, 4°C) and the upper phase was removed and replaced by an artificial upper phase (from MTBE/MeOH/ddH2O, 9/4/4, v/v/v). After a new incubation and centrifugation step, as described above, the complete upper phase was removed and the lower phase was collected and (i) split and dried (for ^13^C analysis) or (ii) dried without splitting (for ^15^N analysis) using a vacuum concentrator. Metabolites for ^13^C and ^15^N analysis were dissolved in 70% acetonitrile (0.5 mM medronic acid) for further LC-MS analysis. Carboxylates for ^13^C analysis were dissolved in 75% MeOH for derivatization. Samples were derivatized by the addition of 30 µL 3-nitrophenylhydrazine (3NPH, 150 mM in MeOH), 30 µL 1-ethyl-3-(3-dimethylaminopropyl)carbodiimide (EDC, 150 mM in MeOH), 30 µL pyridine (7.5% in 75% MeOH) and incubated for 30 min on ice followed by 30 min on 50 °C under shaking. Samples were cooled down on ice and the reaction was stopped by adding 50 µL formic acid (0.98%). Samples were centrifuged (13,000 rpm, 10 min, RT) for further LC-MS analysis. For each analysis (^13^C, ^13^C derivatized, ^15^N), aliquots of all according samples were combined into a pooled quality control sample (QC) which was analyzed at the start and end of the sequence as well as after each fifth sample injection. Two empty extractions (sample-free for background control) were injected before and after sample injections. An in-house reference compound mix (for level 1 identification) was injected at the start of the sequence. Each sample matrix-free injection was followed by two QC injections for column re-equilibration. Protein precipitates of the extraction were dried, solubilized in NaOH (0.3 N, 55 °C, > 4 h) and the protein content was determined using Pierce™ BCA reagent (Thermo Fisher Scientific) according to the manufacturer’s guidelines.

Chromatographic separation was performed on Vanquish systems (Thermo Fisher Scientific) equipped with an ACQUITY UPLC BEH Amide column (2.1 × 150 mm, 1.7 µm; Waters, Milford, MA), using an 18 min gradient (400 µl/min) from 97% solvent A (ACN/ddH2O, 95/5, v/v; 10 mM NH4FA, 10 mM NH3) to 65% solvent B (ddH2O/ACN, 95/5, v/v; 20 mM NH4FA, 20 mM NH3). The column compartment was kept at 40 °C. For ^13^C analysis a QExactive Focus mass spectrometer (Thermo Fisher Scientific) equipped with a heated electrospray ionization (HESI II) source was used for detection of the metabolites in negative data dependent acquisition mode (MS1: m/z 60–900, resolution 70,000, AGC target 1e6, IT auto; MS2: resolution 17,500, isolation window 2 m/z, NCE 30, AGC target 5e4, IT auto). For ^15^N analysis an Orbitrap Eclipse Tribrid mass spectrometer (Thermo Fisher Scientific) equipped with a heated electrospray ionization source (OptaMax NG) was used for detection of the metabolites in negative acquisition mode (MS1: m/z 80–900, resolution 240,000, AGC target 3e5, IT auto). Metabolites were identified either level 1 via accurate m/z of the [M-H]- ion ( < 5 ppm) and comparison of the retention time (rt) and MS2 spectra to synthetical reference compounds or level 2 without reference rt. All peaks (QC) were manually inspected in Freestyle (1.8 SP2) and peak extraction of the monoisotopic peak and regarding isotopes of interest was performed in Skyline (^15^N, 24.1.0.199) or El-Maven (^13^C, v0.12.0). Only metabolites with < 25% peak area variation (monoisotopic) in QC samples were used for further processing. El-Maven software [29] was used for peak quantification and correction for natural abundance was performed with Isocor [30]. Calculated metabolite abundances were normalized to glutarate and total protein amount.

### Quantitative real-time PCR (qPCR)

RNA was extracted using the peqGOLD Total RNA Kit (VWR, Radnor, PA, USA) according to the instructions of the manufacturer. 500–1000 ng of total RNA were reverse transcribed with the qScript cDNA synthesis kit (Quantabio, Beverly, MA, USA). Quantitative PCR (qPCR), was performed on a LightCycler 480 (Roche, Basel, CH) using QuantiFast SYBR PCR kit (Qiagen, Hilden, DE). Primers used were *GOT1*, 5’-AGCTGTGCTTCTCGTCTTGC-3’ (forward), 5’-AGATTGCACACCTCCTACCC-3’ (reverse); *GOT2*, 5’-GACCAAATTGGCATGTTCTGT-3’ (forward), 5’-CGGCCATCTTTTGTCATGTA-3’ (reverse); *SLC1A1*, 5’-AATGCGGATGCTGAAACTCA-3’ (forward), 5’-ACCACCAGCACAATACCTAGA-3’ (reverse); *SLC1A3*, 5’-CGAAGCCATCATGAGACTGGTA-3’ (forward), 5’-TCCCAGCAATCAGGAAGAGAA-3’ (reverse); *ACTB,* 5’-ATTGCCGACAGGATGCAGGAA-3’ (forward), 5’-GCTGATCCACATCTGCTGGAA-3’ (reverse).

### Western blot

Cells were washed with PBS and lysed on ice with RIPA buffer (Sigma-Aldrich) supplemented with protease/phosphatase inhibitors (Thermo Fischer Scientific). Samples (5–10 µg/lane) were separated on a 10% SDS-PAGE gel and transferred to a PVDF membrane (Bio-Rad). After blocking in 5% bovine serum albumin (BSA)-TBST or milk-TBST for 1 hour at room temperature, membranes were probed with GOT1 antibody (ab239487, Abcam, Cambridge, UK; milk) 1:1000, GOT2 antibody (HPA018139, Sigma-Aldrich; milk) 1:2000, or VDAC1/2/3 mitochondrial loading control antibody (ab15895, Abcam; BSA) 1:5000. As a loading control, membranes were incubated with a β-actin antibody (sc-47778, clone C4, Santa Cruz Biotechnology, Dallas, TX) 1:3000 in 5% milk-TBST or with GAPDH-antibody conjugated to peroxidase (sc-365062) 1:5000 in 5% BSA-TBST.

### GOT activity enzymatic assay

Cells (20 Mio) were plated in cell culture flasks and treated with high or low glucose media for 48 hours with medium replacement after 24 hours. After treatment, cells were collected by trypsinization and mitochondria were isolated, as described below. Mitochondria were resuspended in analysis buffer and homogenized by sonication. Protein concentrations were determined using the BCA Protein Assay Kit (Thermo Fisher Scientific). The Aspartate Aminotransferase (AST, GOT) Activity Assay Kit (Colorimetric) (Cell Biolabs, Inc., San Diego, USA) was used to measure GOT activity using a spectrophotometric plate reader (CLARIOstar Plus, BMG Labtech, Ortenburg, DE) according to the manufacturer’s protocol.

### Isolation of mitochondria

Cells were harvested using trypsinization and washed with PBS before centrifugation at 400 g for 5 min. Thereafter, cells were resuspended in 1 mL of ice-cold IMBc buffer (200 mM sucrose, 10 mM Tris/MOPS, 10 mM EGTA/Tris, pH 7.4) and passed 12 times through the Isobiotec Cell Homogenizer (Isobiotech, Heidelberg, Germany) using a 10 µm clearance ball. The homogenate was centrifuged at 600 g for 15 min at 4 °C to remove debris. The supernatant was further centrifuged at 9000 g for 15 min at 4 °C. The supernatant, containing the cytoplasmic fraction, was stored at −80 °C. The pellet, which contained the mitochondria, was resuspended in 300 µL IMBc for an additional washing step and centrifuged again at 9000 g for 15 minutes at 4 °C. Mitochondria were quickly frozen and stored at −80 °C prior further analysis.

### NAD^+^/NADH ratio determination

For determination of the NAD^+^/NADH ratio, 300,000 cells per well were plated onto 6-well plates in complete growth medium and treated as indicated. In rescue experiments, 5 mM lactate or 5 mM pyruvate (Sigma-Aldrich) were added 5 hours prior to sampling. Plates were washed with PBS and immediately frozen on liquid nitrogen to quench metabolism. The NAD^+^/NADH assay was performed in a modified manner according to the manufacturer (NAD/NADH-Glo™ Assays, Promega, Madison, USA) [31]. For extraction, plates were washed quickly with PBS on ice, lysed with ice-cold extraction buffer (PBS/1% dodecyltrimethylammonium bromide (DTAB) in 0.2 M NaOH (1/1) (v/v)) and processed according to the manufacturer. Luminescence was measured using the CLARIOstar Plus. The NAD^+^/NADH ratio was calculated by dividing the luminescence signal of separately prepared acid treated samples (NAD^+^) by the signal of samples in alkaline conditions (NADH).

### Proliferation assays

For the assessment of proliferation rate, the Click-iT EdU Alexa Fluor 488 (or Pacific Blue) Flow Cytometry Assay Kit (Invitrogen) was used according to the manufacturer’s instructions. Briefly, cells were cultured in 6-well plates at a density of 300,000 cells/well in growth medium. After treatment with the respective media for 24 hours, 10 µM of EdU was added for 1.5 hours at 37 °C. Cells were analyzed following the Click-iT reaction containing the fluorescent dye azide by flow cytometry (CytoFlex, Beckman Coulter, Barea, California, USA).

### Gene expression analysis using publicly available datasets

Gene expression data of MAS genes were retrieved from the publicly available TCGA lung adenocarcinoma (LUAD) dataset using the UCSC Xena platform (https://xenabrowser.net/). TCGA gene expression data were generated by the TCGA Research Network (https://www.cancer.gov/tcga).

### Statistics

Data were compiled and analyzed with the software package SPSS, version 29.0 (Chicago, IL) or with Prism 10 (Graph Pad, Boston, MA). Group differences were calculated using Student´s t-test, one group Student´s t-test, One-way ANOVA or Two-way ANOVA with Tukey, Dunnett or Sidak´s post-hoc analysis, or Mann-Whitney U test as appropriate. *P*-values smaller than 0.05 were considered significant.

## Results

### Aspartate *de novo* synthesis and release are enhanced in low glucose conditions

**Fig. 1:**
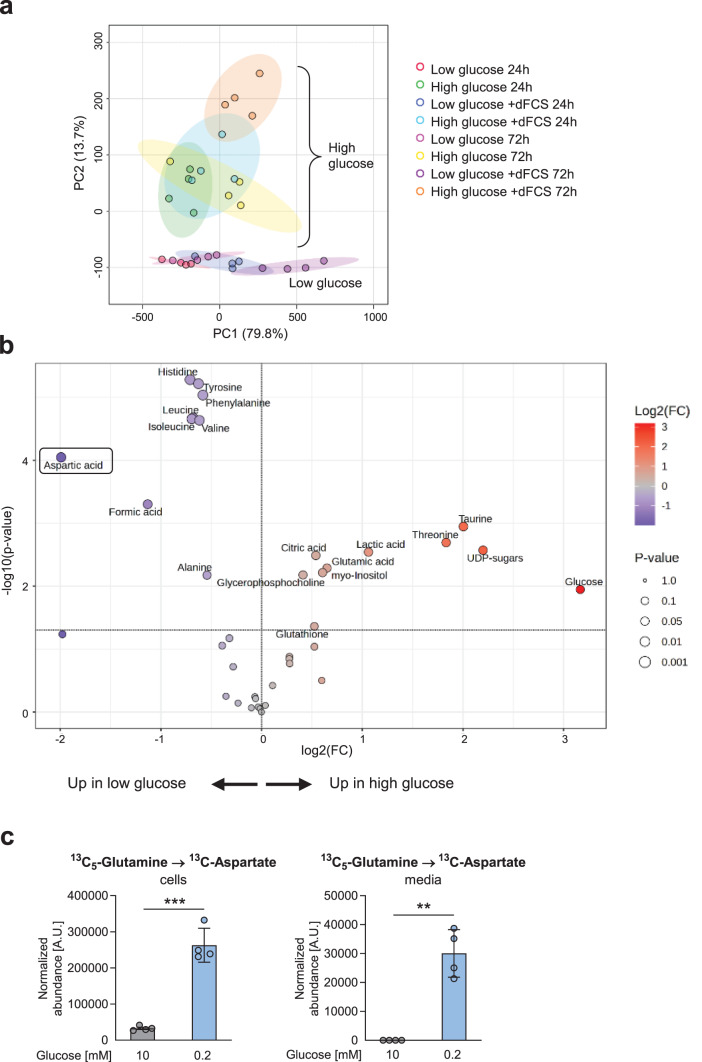
NMR spectroscopy reveals an accumulation and release of aspartate in glucose-deprived lung cancer cells. (**a**) PCA of metabolomes analyzed by ^1^H NMR spectroscopy in A549 cells cultured in high (10 mM) or low (0.2 mM) glucose conditions with or without 10% dialyzed FCS (dFCS) for different time intervals with daily medium change. (**b**) Volcano plot showing significantly changed metabolites. (**c**) ^13^C NMR spectroscopy shows significantly enhanced labeled aspartate (Hβ-Cβ) from ^13^C_5_-glutamine in cells and media supernatants after 24 hours. Data are shown as mean ± SEM from four independent experiments. **p < 0.01; ***p < 0.001 on students t-test (cells) or one group students t-test (media)

To study global changes in cancer cell metabolism in low glucose conditions, we performed nuclear magnetic resonance (NMR) spectroscopy in combination with stable isotopic labeling in A549 lung cancer cells. Cells were treated with a physiological high level of glucose (10 mM) compared to low glucose (0.2 mM) conditions in the presence or absence of dialyzed serum and ^13^C_5-_glutamine. Principal component analysis (PCA) revealed a clear clustering according to administered glucose levels (Fig. 1a). Surprisingly, aspartate was among the most highly enriched metabolites in low glucose conditions (Fig. 1b). ^13^C-NMR revealed a clear increase in ^13^C-labeled aspartate under glucose deprivation, showing that aspartate was *de novo* synthesized from glutamine (Fig. 1c). Furthermore, we found an accumulation of labeled aspartate in the conditioned media (Fig. 1c).

**Fig. 2:**
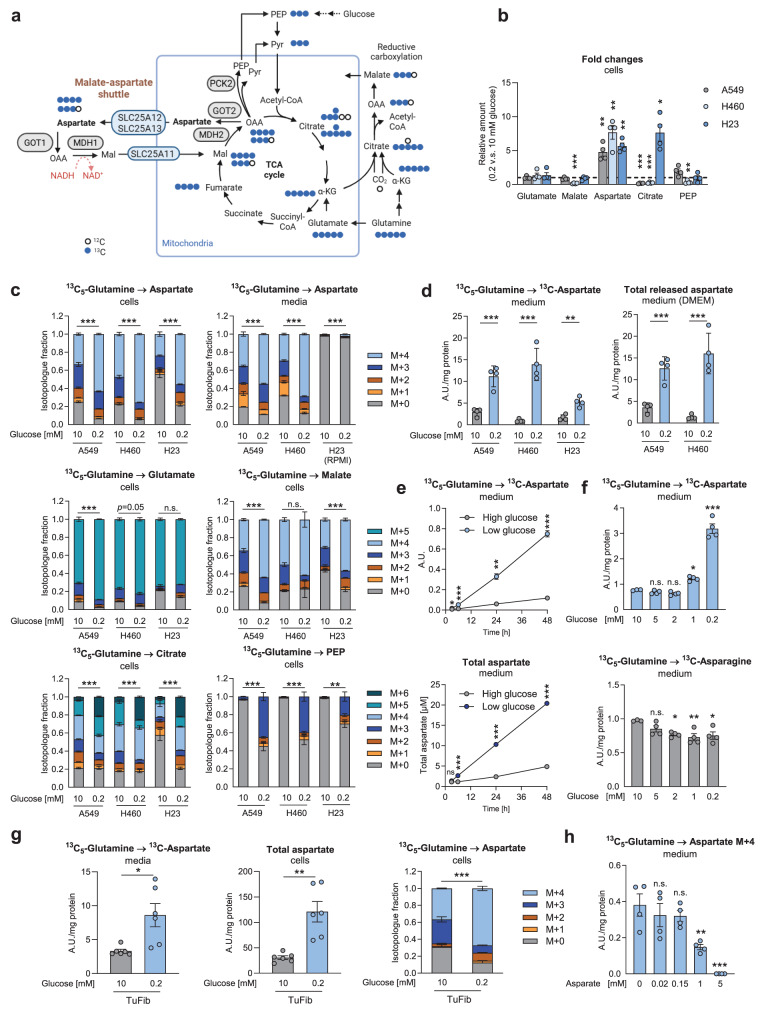
Aspartate release and alterations in central carbon metabolism in low glucose conditions. (**a**) Metabolic downstream pathways of ^13^C_5_-glutamine. (**b–d**) Uniformly labeled [^13^C_5_]-glutamine was administered to different cancer cells in serum-free media containing high (10 mM) or low (0.2 mM) glucose after 24 hours of pre-conditioning in the respective media containing unlabeled glutamine. Metabolites were quantified by GC-MS. (**b**) Fold changes of total metabolite levels (normalized to protein) in 0.2 mM glucose v.s. 10 mM glucose. The dashed line indicates fold change = 1 (no change). (**c**) Label enrichments in aspartate, TCA cycle metabolites and PEP. M + 0 denotes unlabeled metabolites, M + 1, M + 2 and so on, contain one, two, or more ^13^C. (**d**) Release of ^13^C-labeled (left) or total aspartate (right) to the media supernatant within the 24 hours labeling period. For total aspartate release, only cells cultured in medium lacking aspartate (DMEM) are shown, while H23 cells are treated with RPMI containing high basal levels of (unlabeled) aspartate. (**e**) Labeled aspartate in media supernatants of A549 cells treated with ^13^C_5_-glutamine after different time points of low glucose treatment (top) and total medium aspartate levels (bottom). (**f**) Dependency of labeled aspartate release on glucose concentrations. (**g**) Enrichment, total aspartate abundance and release of ^13^C-labeled aspartate in ^13^C_5_-glutamine treated cancer-associated fibroblasts in media lacking dFCS. (**h**) Release of labeled aspartate in ^13^C_5_-glutamine treated A549 cells supplied with different concentrations of exogenous aspartate. Data are shown as mean ± SEM from four (**b–f,h**) or six (**g**) independent experiments. Group comparisons were performed by Student’s t-test (**c,d,g**), one group Student’s t-test v.s. no change (value = 1) (**b**), two-way ANOVA with Sidak’s post-hoc analysis (**e**) or one-way ANOVA with Dunnett post-hoc analysis (**f,h**). (**c,g**) Statistical analysis for isotopologue fractions was performed on relative enrichment of the highest isotopologue. *p < 0.05; **p < 0.01; ***p < 0.001; n.s., not significant. TCA cycle, tricarboxylic acid cycle; PEP, phosphoenolpyruvate; PCK2, phosphoenolpyruvate carboxykinase mitochondrial isoform; GOT1, cytoplasmic aspartate aminotransferase; GOT2, mitochondrial aspartate aminotransferase; TuFib, tumor fibroblasts (cancer-associated fibroblasts)

In order to investigate the metabolic adaptations in central carbon metabolism in different cell types, we performed stable isotopic labeling followed by GC-MS in lung cancer cell lines (A549, H460 and H23), normal primary bronchial epithelial cells as well as cancer-associated fibroblasts. The labeling patterns of incorporated ^13^C_5_-glutamine are depicted in Fig. 2a. Media lacking dialyzed serum were used in all consecutive experiments. Similar to the NMR metabolomics approach, we found significantly enhanced levels of aspartate in all cancer cell lines under low glucose conditions (Fig. 2b), while total levels of the TCA cycle intermediates malate and citrate were inconsistently changed (Fig. 2b). Likewise, the fractional enrichment of fully ^13^C-labeled aspartate (M + 4, denoting four ^13^C) was increased (Fig. 2c). Enhanced fully labeled fractions of malate (M + 4) suggest an overall increased contribution of glutamine to the TCA (Fig. 2c). Similar to the NMR metabolomics experiment, we found a clearly increased release of labeled and total aspartate to the medium in low versus high glucose (Fig. 2d).

In the case of A549 and H460 cells, isotopologue distributions of aspartate were similar in cells and media, since these are supplied with DMEM, which lacks aspartate, and M + 4 was the most frequent isotopologue (Fig. 2c). Accordingly, in H23 cells cultured in RPMI, which contains 150 µM aspartate, the frequencies of the labeled isotopologues in the media supernatants were considerably lower (Fig. 2c). The accumulation of labeled aspartate was time-dependent (Fig. 2e, top). When we analyzed total medium aspartate in A549 cells, levels reached 20 µM under low glucose conditions after 48 hours, which is roughly the concentration found in human serum [32] (Fig. 2e, bottom). Assessing the glucose-dependency of labeled aspartate release in ^13^C_5_-glutamine treated A549 cells, elevated levels of labeled aspartate were noted at 1 mM and 0.2 mM glucose compared to 10 mM, while labeled asparagine, an amino acid generated from aspartate, was found slightly decreased by glucose deprivation (Fig. 2f).

An accumulation and release of aspartate was also found in cancer-associated fibroblasts isolated from primary non-small cell lung cancers, the main histological subtype of lung cancer (Figs. 2g and S1a–c) and in bronchial epithelial cells (Fig. S1d). Of note, physiological levels of exogenous aspartate did not affect the release of labeled aspartate generated from ^13^C_5_-glutamine under low glucose conditions but high, supraphysiological levels of 1 mM and 5 mM aspartate inhibited aspartate export, suggesting an impact on transport kinetics (Fig. 2h). Citrate M + 6 fractions, resulting from condensation of fully labeled OAA (M + 4) with labeled acetyl-CoA (M + 2), were increased in all cancer cell lines under low glucose conditions (Fig. 2c). Glutamine conversion to glutamate was inconsistently changed, a clear increase of labeled glutamate was found in A549 cells (Fig. 2c) while glutamate M + 5 was decreased in fibroblasts (Fig. S1c). Interestingly, all cancer cell lines and fibroblasts exhibited reductive carboxylation, as shown by malate/aspartate M + 3 labeling (Figs. 2c, g and S1c) and citrate M + 5 labeling (assessed in the cancer cells, Fig. 2c), however, it was variably modulated by low glucose conditions.

The partial gluconeogenesis pathway, mediated by phosphoenolpyruvate carboxykinase (PCK2), has been shown previously by us and others to be activated in cancer cells under low glucose conditions, allowing the synthesis of glycolytic intermediates from non-carbohydrate precursors (pathway map, Fig. 2a) [33–38]. In order to clarify, whether the TCA cycle intermediate OAA is directed to the initial steps of gluconeogenesis, we assessed the labeling of the glycolytic/gluconeogenic intermediate phosphoenolpyruvate (PEP) from ^13^C_5_-glutamine. Indeed, the label was transferred to PEP in low but not in high glucose conditions (Fig. 2c), showing the activation of partial gluconeogenesis. Together the results indicate that aspartate is produced and released in glucose limited cancer cells and different non-neoplastic cells. Additionally, glucose-deprived cells show enhanced glutamine contribution to the TCA cycle and activation of partial gluconeogenesis.

### GOT2 is responsible for aspartate synthesis

**Fig. 3:**
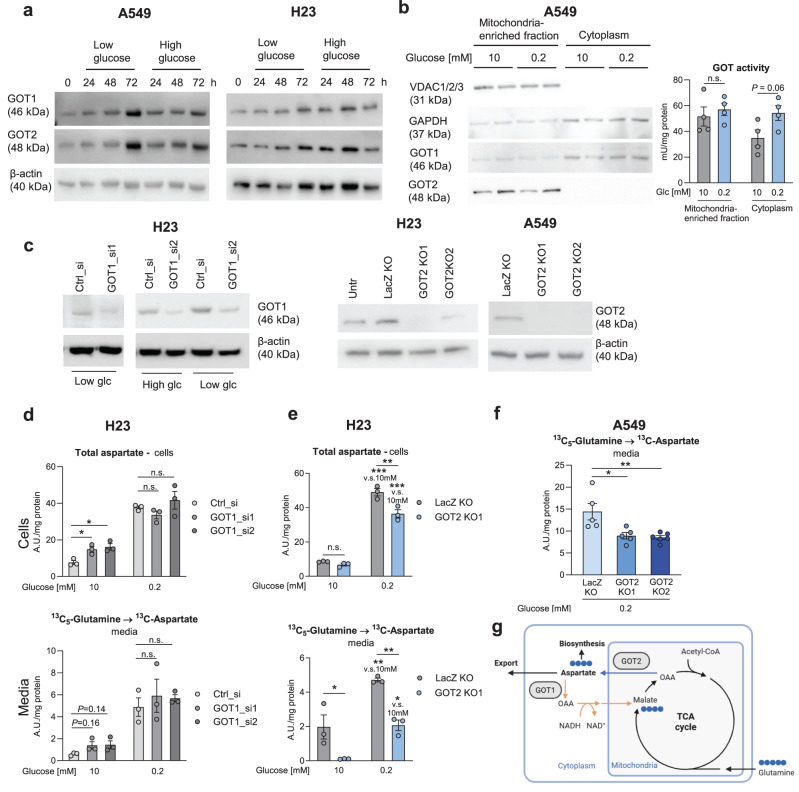
GOT2 is responsible for aspartate synthesis. (**a**) Expression of GOT1 and GOT2 in cancer cells under different conditions. Representative Western blots are shown. (**b**) GOT activity measured by an enzymatic assay in mitochondria-enriched and cytoplasmic fractions obtained from A549 cells treated with high or low glucose conditions for 48 hours. VDAC1/2/3 and GAPDH Western blots as controls for mitochondria and cytoplasm, respectively, are shown. (**c**) Silencing efficiencies of GOT1 silencing by siRNA pools (GOT1_si1 and si2) and of GOT2 knockout (GOT2 KO1 versus LacZ KO). Representative Western blots are shown. (**a,c**) β-actin was used as a loading control. (**d-f**) Impact of or GOT1 silencing or CRISPR-Cas9 mediated GOT2 knockout (**d,e**) on cellular aspartate and labeled aspartate release from^13^C_5_-glutamine (**a-f**) serum-free media were used. Results are displayed as mean ± SEM from four (**b**), six (**d**), or three (**e,f**) independent experiments. Group comparisons were performed by Student’s t-test (**b,d**), two-way ANOVA with Tukey post-hoc analysis one-way ANOVA with Dunnett post-hoc analysis (**f**). *p < 0.05; **p < 0.01; ***p < 0.001; n.s., not significant. (**g**) Schematic for GOT1- and GOT2-mediated aspartate metabolism within the MAS. The proximal and distal arm of the MAS are highlighted in blue and orange, respectively. Blue circles denote ^13^C. TCA cycle, tricarboxylic acid cycle

Next, we aimed to address the metabolic route leading to *de novo* aspartate generation. Both, GOT1, GOT2 showed enhanced expression in lung adenocarcinoma (LUAD) compared to normal lung in the publicly available TCGA LUAD dataset (Fig. S2). Similarly, other components of the MAS, MDH1 and MDH2, as well as the aspartate–glutamate carriers SLC25A12 (aralar) and SLC25A13 (citrin), were significantly overexpressed in the cancer tissue, while SLC25A11, the malate carrier, was down-regulated (Fig. S2). Different glucose concentrations did not significantly impact the protein levels of GOT1 or GOT2 in lung cancer cells (Fig. 3a, quantification in Fig. S3a). Likewise, GOT activity was not altered by different glucose levels in the mitochondria-enriched fractions, however, a trend for enhanced GOT activity in the cytoplasmic fractions of low v.s. high glucose-treated cells was found (Fig. 3b). Of note, cytoplasmic fractions were mitochondria-free, as shown by the absence of non-selective voltage-gated ion channel (VDAC) 1/2/3, a common mitochondria marker, and thus contained only GOT1 (Fig. 3b). However, mitochondrial fractions, collected by an established but rather gentle dissociation protocol, contained also minor levels of cytoplasmic proteins (Fig. 3b). In order to address the role of GOT1 versus GOT2 in aspartate synthesis, we utilized siRNA mediated silencing (GOT1) or a CRISPR-Cas9 mediated gene editing approach (GOT2) (Figs. 3c and S3b). CRISPR-Cas9 was used to induce GOT2 knockout since GOT2 siRNA did not achieve sufficient silencing. In H23 cells, total cellular aspartate levels were increased by two different GOT1 siRNA pools in high glucose conditions, but not in low glucose media (Fig. 3d). Moreover, GOT1 siRNA showed a trend of increased aspartate release in high, but not in low glucose conditions (Fig. 3d). These data suggest that GOT1 mediates aspartate metabolism in high glucose levels, as would be expected in highly glycolytic cells engaging the canonical MAS. However, GOT1 silencing did not further modulate aspartate levels in low glucose, suggesting a diminished activity of the “distal” part of the MAS under low glucose (scheme shown in Fig. 3g, orange arrows). In contrast, GOT2 knockout led to reduced aspartate release to the medium in both, high and low glucose conditions in two different cell lines (Fig. 3e,f), showing that GOT2 was responsible for aspartate generation in both conditions and that the initial steps (“proximal arm”) of the MAS are still activate under low glucose availability (Fig. 3g, blue arrow). Total cellular aspartate levels were likewise reduced by GOT2 knockout, yet the decrease was milder and not significant in high glucose conditions, possibly due to homeostatic mechanisms (Fig. 3e). Taken together, GOT2 was responsible for aspartate synthesis, both, under low and high glucose conditions.

### A decline in NADH contributes to aspartate release in low glucose conditions likely by suppressing the downstream steps of the MAS

**Fig. 4:**
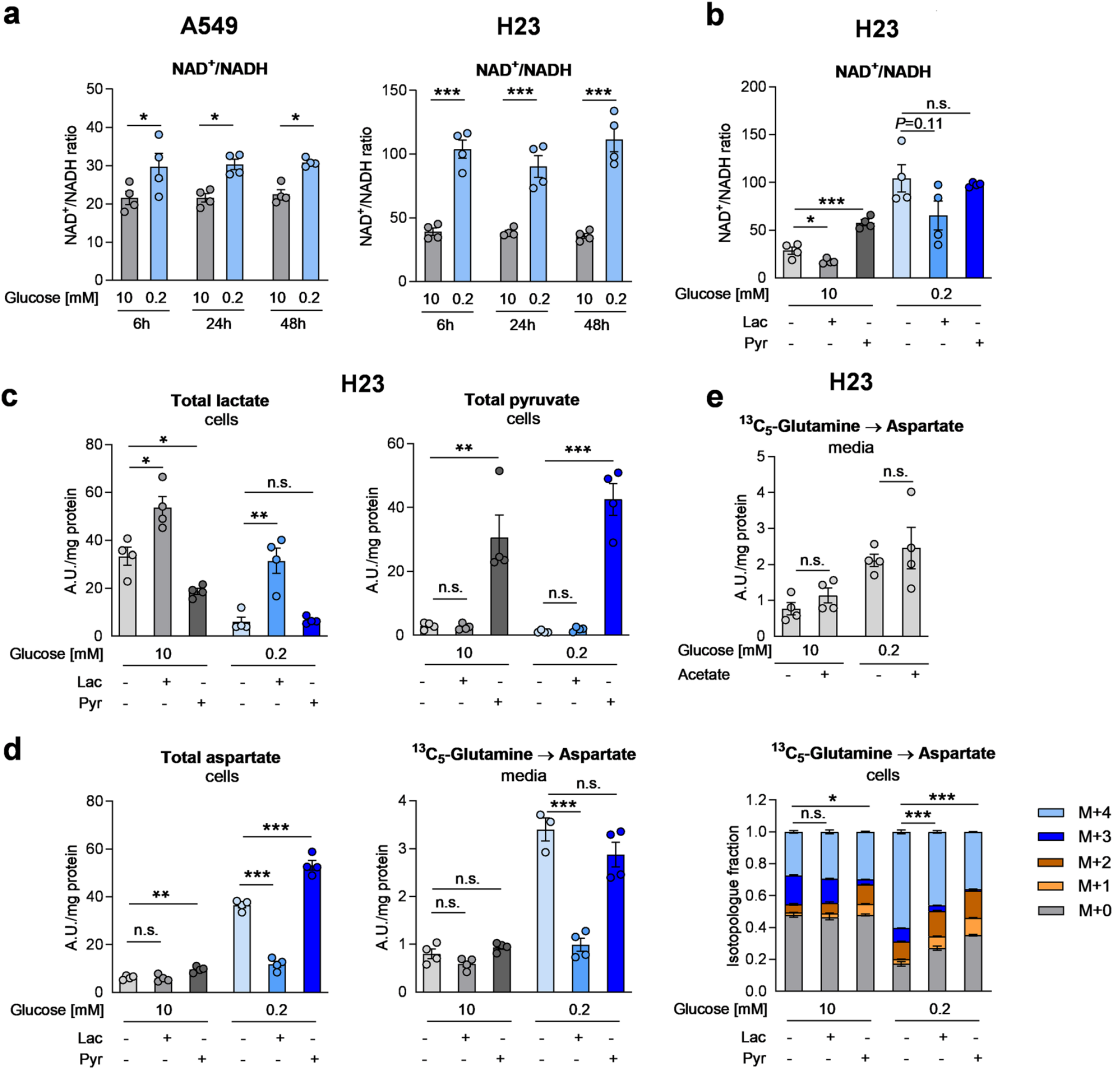
A decline in NADH contributes to aspartate release in low glucose conditions. (**a**) Time-course of NAD^+^/NADH ratio measured enzymatically in high or low glucose conditions in serum-free media. (**b**) NAD^+^/NADH ratio in lactate or pyruvate treated H23 cells. (**c**) Changes in lactate and pyruvate levels in lactate or pyruvate treated H23 cells. (**d,e**) total aspartate levels, aspartate enrichment and labeled aspartate release in ^13^C_5_-glutamine treated H23 cells supplemented with 5 mM lactate, 5 mM pyruvate or 0.5 mM acetate in media lacking dFCS. (**a-e**) Results are displayed as mean ± SEM from four experiments. Group comparisons were performed by two-way ANOVA with Sidak’s post-hoc analysis (**a**), Student’s t-test (**c-e**) or one-way ANOVA with Dunnett post-hoc analysis (**b**). (**d**, right) Statistical analysis was performed on relative enrichment of the highest isotopologue. *p < 0.05; **p < 0.01; ***p < 0.001; n.s., not significant

Low glucose supplementation reduces glycolysis and thus the conversion of NAD^+^ to NADH in the GAPDH step of glycolysis and by downstream pyruvate dehydrogenase. Indeed, the NAD^+^/NADH ratio increased under low glucose conditions (Fig. 4a). In order to investigate, whether the decline in NADH modulates the MAS and thereby aspartate synthesis and release, we utilized exogenous lactate, which generates NADH intracellularly upon oxidation to pyruvate, or exogenous pyruvate, which consumes NADH generating NAD^+^. The NAD^+^/NADH ratio (Fig. 4b) and the total cellular levels of lactate and pyruvate (Fig. 4c) were changed in an expected manner, yet pyruvate modulated NAD^+^/NADH only in high glucose conditions. The reason is unknown but a depletion of NADH under low glucose or a direct use of pyruvate in metabolism potentially play a role. Importantly, exogenous lactate rescued the aspartate accumulation in the cells under low glucose conditions (Fig. 4d). Additionally, lactate completely suppressed low-glucose enhanced ^13^C-aspartate release (Fig. 4d). The effect was not attributable to different aspartate labeling ratios. In fact both, lactate and pyruvate slightly suppressed aspartate M + 4 enrichment (Fig. 4d). This might potentially be attributable to a replenishing of the TCA cycle in an anaplerotic manner. When we added acetate as an additional carbon source not directly modulating NAD^+^/NADH, no significant impact on aspartate release was found (Fig. 4e). Together these data show that the NADH-generating agent lactate almost completely reversed low-glucose enhanced aspartate release, potentially by facilitating the MDH1 reaction in the “distal” MAS.

### Hypoxia reduces aspartate accumulation in lung cancer cells

**Fig. 5:**
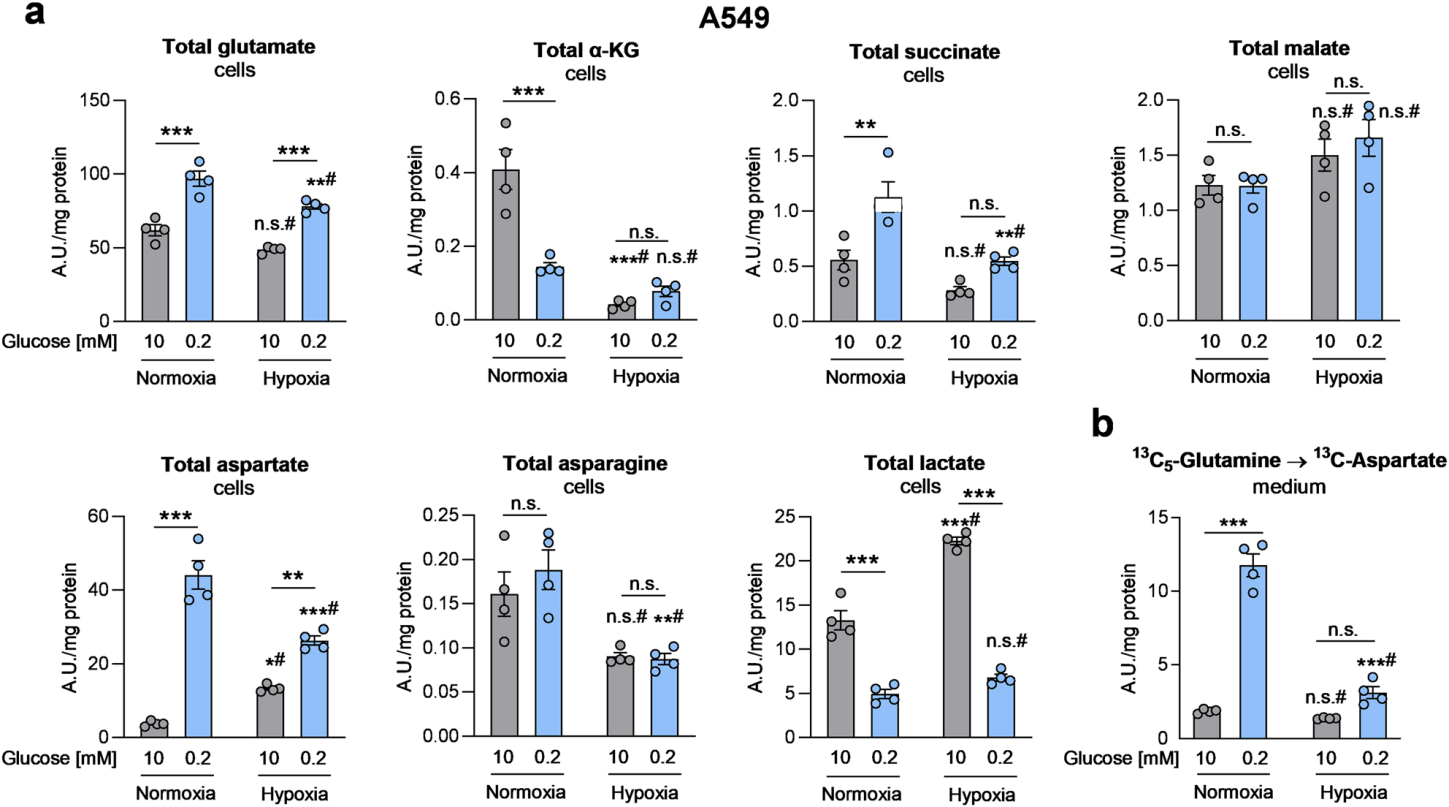
Hypoxia reduces aspartate accumulation in lung cancer cells. A549 cells were treated with serum-free, high or low glucose media in normoxia (ambient oxygen, 21%) or in hypoxia (0.5%) for 24 hours, followed by ^13^C_5_-glutamine labeling for additional 24 hours at the respective oxygen and glucose conditions. (**a**) Cellular total levels of different TCA cycle metabolites, aspartate and lactate. (**b**) Release of labeled aspartate to the medium. (**a,b**) Data are shown as mean ± SEM from four independent experiments. Group comparisons were performed using two-way ANOVA with Tukey post-hoc analysis. *p < 0.05; **p < 0.01; ***p < 0.001; n.s., not significant; ^#^ versus normoxia

Next, we analyzed the effects of hypoxia, since cancer cells treated with hypoxia or electron transport chain inhibitors have been shown to exhibit suppressed aspartate synthesis [13, 39]. In A549 cells, hypoxia reduced aspartate levels only under low glucose conditions, while aspartate was slightly elevated under hypoxia in high glucose conditions (Fig. 5a). Glucose deprivation led to an increase of aspartate abundance in both oxygen conditions (Fig. 5a). Low glucose-enhanced aspartate levels (Fig. 5a) and release to the medium (Fig. 5b) were partly suppressed by hypoxia. Of note, hypoxia led to enhanced reductive carboxylation, under high, but not low glucose conditions, as suggested by elevated malate and aspartate M + 3 fractions (Fig. S4). The enhanced reductive carboxylation under hypoxia, via the reverse isocitrate dehydrogenase reaction, is in line with previously published reports [40, 41]. In summary, hypoxia reduced the low glucose induced accumulation and release of aspartate.

### Reduced utilization of aspartate in purine synthesis under low glucose conditions

**Fig. 6:**
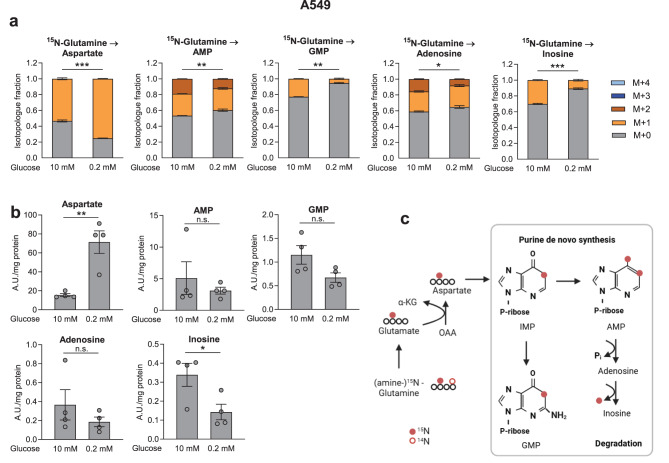
Low glucose treatment reduces aspartate contribution of nitrogen to purine synthesis. (**a,b**) A549 cells were treated with the respective glucose levels for 24 hours and treated with the same media containing (amine-)^15^N-glutamine for additional 24 hours. Cells were analyzed by liquid chromatography - mass spectrometry. (**a**) Isotopologue fractions and (**b**) total abundance of purines. (**a,b**) Results are displayed as mean ± SEM from four independent experiments. Statistical analysis was performed on the relative enrichment of M + 3. Group comparisons were performed by Student’s t-test. *p < 0.05; **p < 0.01; ***p < 0.001; n.s., not significant; (**c**) labeling scheme for the transfer of the (amine-)^15^N-glutamine label. IMP, inosine monophosphate; AMP, adenosine monophosphate; GMP, guanosine monophosphate

Aspartate fuels diverse downstream pathways. In cancer cells, the provision of nitrogens for purine synthesis is key, thus we investigated the effects of low glucose supplementation on purine synthesis using amine-^15^N-glutamine. The nitrogen is transferred onto aspartate by GOT giving rise to ^15^N-labeled purines (scheme in Fig. 6c). In A549 cells, labeled fractions in aspartate increased, in line with increased *de novo* synthesis, however, purine ^15^N labeling was significantly reduced (Fig. 6a) along with a reduction of inosine and a trend for reduced purine abundance (Fig. 6b). This suggests diminished purine *de novo* synthesis rates, either due to a lower demand in slowly proliferating cells or due to limitations in other precursors, such as ribose-phosphate. Thus, a reduced utilization of aspartate in purine nucleotide synthesis may contribute to increased aspartate levels under low glucose conditions. Another metabolic route for aspartate, aspartate to asparagine conversion, showed inconsistent changes in the different cell lines. Labeling of asparagine from ^13^C glutamine paralleled changes in aspartate in A549 and H460 cells, although the asparagine pool size was partly reduced (Fig. S1e), making an interpretation difficult. H23 cells, grown in RPMI which contains 380 µM asparagine, showed extremely low asparagine labeling, suggesting that these cells import it from the medium (Fig. S1e).

### Gluconeogenesis proceeds via the NADH-dependent reverse GAPDH reaction and contributes to the generation of ribose-5-phosphate

**Fig. 7:**
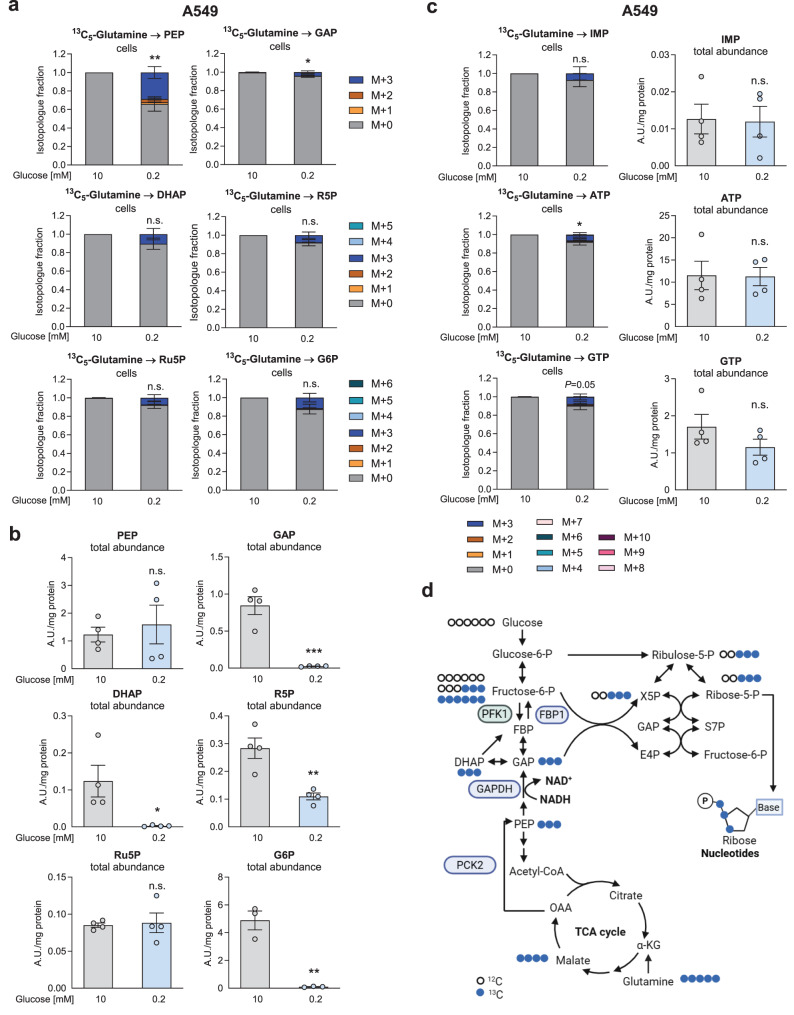
Low glucose treatment activates partial gluconeogenesis. (**a-c**) A549 cells were treated with the respective glucose levels for 24 hours and treated with the same media containing ^13^C_5_-glutamine for additional 24 hours. Cells were analyzed by liquid chromatography - mass spectrometry. (**a**) Isotopologue fractions and (**b**) total abundance of glycolysis/gluconeogenesis and pentose phosphate pathway metabolites. (**c**) isotopologue fractions and abundances of purines. (**a-c**) Results are displayed as mean ± SEM from four independent experiments. Statistical analysis was performed on the relative enrichment of M + 3. Group comparisons were performed by Student’s t-test or one-group Student’s t-test as applicable. *p < 0.05; **p < 0.01; n.s., not significant; (**d**) labeling scheme for ^13^C_5_-glutamine-derived partial gluconeogenesis and ribose-5-phosphate synthesis. PEP, phosphoenolpyruvate; GAP, glyceraldehyde-3-phosphate; R5P, ribose-5-phosphate; Ru5P, ribulose-5-phosphate; G6P, glucose-6-phosphate; X5P, xylulose-5-phosphate; IMP, inosine monophosphate; AMP, adenosine monophosphate; GTP, guanosine triphosphate, GAPDH, glyceraldehyde-3-phosphate dehydrogenase; PCK2, phosphoenolpyruvate carboxykinase mitochondrial isoform; PFK1, phosphofructokinase 1; FBP1, fructose-1,6-bisphosphatase 1

While the canonical glycolytic GAPDH reaction produces NADH from NAD^+^, thereby converting glyceraldehyde-3-phosphate (GAP) to 1,3-bisphosphoglycerate, the reverse reaction takes place in gluconeogenesis. In order to clarify whether (partial) gluconeogenesis proceeds through the reverse GAPDH reaction in low glucose conditions, we utilized uniformly ^13^C-labeled glutamine and assessed the label transfer to upper glycolytic and pentose phosphate pathway (PPP) intermediates. The PPP is a chain of reactions fueled by glucose-6-phosphate (oxidative arm) or fructose-6-phosphate and GAP (non-oxidative arm; scheme in Fig. 7d). Strikingly, ^13^C_5_-glutamine led to consistent M + 3 labeling of GAP, dihydroxyacetone phosphate (DHAP), ribose-5-phosphate, ribulose-5-phosphate and glucose-6-phosphate in A549 cells (Fig. 7a). Higher labeling of glucose-6-phosphate was not found. Ribose-5-phosphate M + 3 can be generated from GAP M + 3 via the transketolase reaction or from labeled fructose-6-phosphate or glucose-6-phosphate (Scheme in Fig. 7d). The labeled ribose-5-phosphate (M + 3) was incorporated into the nucleotides inosine monophosphate (IMP), adenosine triphosphate (ATP) and guanosine triphosphate (GTP) (Fig. 7c). In H23 cells, PEP labeling from ^13^C_5_-glutamine was clearly observed, however, labeling of ribose-phosphate was not found and the labeling of GAP was rather low (Fig. S5a). These data suggest that initial gluconeogenesis via PCK2, but only a minor fueling of distal gluconeogenesis via GAPDH occurred. The total abundance of glycolytic/gluconeogenic intermediates or PPP intermediates was only partially reduced under low v.s. high glucose conditions (Figs. 7b and S5b), and changes in purine pools were rather mild (Figs. 7c, 6b). Together, these data show that partial gluconeogenesis may proceed beyond the level of GAPDH in a cell-line dependent manner. Thus, NADH may be consumed in the reverse GAPDH direction in partial gluconeogenesis.

### Aspartate serves as a carbon source in a cell-line dependent manner

When we added ^13^C_4_-labeled aspartate exogenously at the concentration present in RPMI medium (150 µM) we found no incorporation into TCA cycle metabolites or asparagine in A549 cells (Fig. S6), despite avid asparagine *de novo* synthesis from endogenous aspartate (Fig. S4). Furthermore, exogenous aspartate was not utilized for purine synthesis in A549 cells in any condition as shown by using ^15^N-labeled aspartate (Fig. S7). However, in H460 lung adenocarcinoma cells, ^13^C_4_-aspartate contributed to asparagine and malate pools to a low extent (Fig. S6a). Interestingly, a higher expression level of the known aspartate transporter SLC1A3 in H460 compared to A549 cells was observed (Fig. S6b), in line with recent studies which showed very low but detectable levels of SLC1A3 in H460 cells [42] and the absence of SLC1A3 in A549 cells [13]. However, the opposite distribution was found for another aspartate transporter, SLC1A1 (Fig. S6b). Thus, aspartate released from cells exposed to low glucose conditions may potentially be used as a carbon source by other cells, yet our data suggest that cancer cells preferably synthesize aspartate *de novo*. Previous studies suggested an important role of exogenous aspartate as nitrogen donor for purine synthesis in cells with a defective electron transport chain [13, 14].

### Low glucose-driven aspartate release does not impact cell proliferation under glucose deprivation

**Fig. 8:**
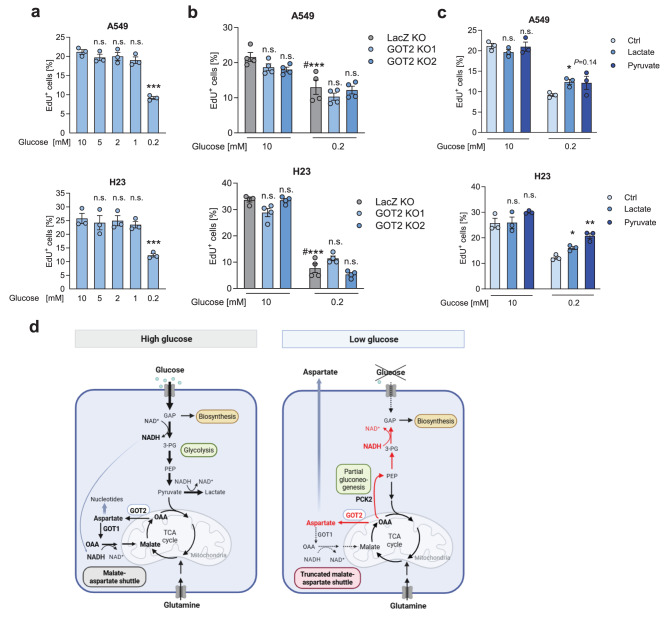
Low glucose- driven aspartate release does not impact cell proliferation under glucose deprivation. (**a**) Cells were treated with serum-free media containing different concentrations of glucose for 24 hours. The fraction of EdU positive (proliferating) cells determined by FACS is shown. (**b**) GOT2 KO cells or control cells were treated with high or low glucose media for 24 hours. (**c**) Cells were treated with high or low glucose media containing 5 mM lactate or pyruvate for 24 hours. Results are mean/± SEM from three (**a,c**) or four (**b**) independent experiments. Group comparisons were performed by one way ANOVA with Dunnett post-hoc analysis (**a**), two-way ANOVA with Tukey post-hoc analysis (**b**) or Student’s- t-test (**c**). (**b**) n.s., not significant versus LacZ; #, v.s. high glucose. (**c**) group comparisons were performed v.s. Ctrl. *p < 0.05; **p < 0.01; ***p < 0.001; n.s., not significant. (**d**) Model for metabolic adaptations under low glucose conditions

Glucose reduction up to 1 mM did not affect cell proliferation. However, diminishing glucose levels to 0.2 mM reduced cell proliferation, as assessed by EdU incorporation assays, after 24 hours of treatment (Fig. 8a). In order to assess the role of aspartate production and release for cell proliferation we measured EdU incorporation in GOT2 knockout cells and lactate/pyruvate treated cells. GOT2-knockout had no consistent effect on cell proliferation, neither in high, nor in low glucose conditions (Fig. 8b). The high rate of OAA to aspartate conversion and further use in asparagine and nucleotide synthesis, as found in the tracing experiments, suggest that GOT1 might compensate and ensure the synthesis of aspartate, as previously shown [39]. Interestingly, under low glucose conditions both, lactate and pyruvate partly rescued cell proliferation after 24 hours (Fig. 8c). This finding argues against a major role of NADH balance for proliferation under these conditions. Rather, the enhanced proliferation by lactate and pyruvate may occur since both can serve as a carbon and/or energy source [33, 43].

## Discussion

Our study shows that low glucose availability results in aspartate release from tumor cells and non-neoplastic cells. We found that the initial reactions of the MAS are still active under low glucose conditions, leading to aspartate *de novo* synthesis in the mitochondria. However, the subsequent reactions of the MAS are impaired due to a decline in NADH, greatly contributing to the accumulation and export of newly synthesized aspartate (model in Fig. 8d). Thus, while the MAS plays a role in maintaining a favorable NAD^+^/NADH ratio to promote glycolysis in proliferating cells [44], glucose shortage diminishes NADH and modulates the MAS leading to aspartate accumulation and release.

The finding that aspartate is released in a condition of carbon (glucose) starvation shows that cell-autonomous regulation balances nutrient availability and central carbon metabolism in cancer and normal cells. Truncation of the MAS might prevent further NADH loss under glucose deprivation. Instead of being shuttled to the mitochondria via the MAS, NADH can be used in NADH-dependent partial gluconeogenesis, as we found, or other NADH-requiring pathways. It has been shown that the activity of MDH1, which mediates the cytosolic conversion of OAA to malate in the “distal arm” of the MAS, in fact requires NADH binding for catalysis [45]. A knockout of GOT1, MDH1, or the mitochondrial oxoglutarate-malate carrier (SLC25A11) responsible for malate import into the mitochondria, all led to clear increases of total cellular aspartate levels in human embryonic kidney cells in a recent study [46], in line with our observed enhancement of aspartate in low glucose/NADH conditions.

Aspartate is required for nucleotide synthesis and impeding the export of *de novo* synthesized aspartate from the mitochondria leads to cancer cell death when glutamine supply is low [47]. Accordingly, respiration-deficient cancer cells with impaired aspartate synthesis require exogenous aspartate [14, 15]. Furthermore, aspartate plays multifaceted roles as an extracellular signaling molecule by acting as an agonist at the N-methyl-D-aspartate (NMDA) receptor [48], a neurotransmitter receptor. In addition to its role in the central nervous system, the NMDA receptor has been implicated in regulation of the vascular tone and in immune cell functions [49–51]. It has been shown that aspartate accumulating in lung interstitial fluid upon pre-metastatic priming by breast cancer cells enhances the aggressiveness of metastases via the NMDA receptor on cancer cells [52]. Thus, the released aspartate may affect neighbouring cells. Potentially, aspartate may support the metabolism of aspartate-auxotrophic cells, given that they express the respective transporters. A549 lung cancer cells and pancreatic cancer cells exposed to hypoxia have been shown to exhibit reduced levels of aspartate and diminished proliferation, which could be rescued by overexpression of a cellular aspartate transporter SLC1A3, allowing aspartate uptake [13]. In our study, hypoxia at similar levels (0.5%) clearly reduced, but did not abrogate aspartate *de novo* synthesis and release. However, hypoxia clearly diminished aspartate release under low glucose conditions.

The role of the exchange of aspartate between different cell types in tumors is still poorly understood. In our study, the H460 cell line showed a small but detectable uptake and conversion of ^13^C-labeled aspartate to asparagine and TCA cycle metabolite, which might be attributable to higher SLC1A3 aspartate transporter expression. When both, GOT1 and GOT2 were deleted, pancreatic cancer cells could be grown in vitro only in media supplemented with supraphysiological levels of aspartate [39]. Interestingly, in vitro cultured cancer cells have been previously shown to exhibit a net aspartate release and independence of exogenous aspartate, suggesting that highly proliferative cells exhibit *de novo* aspartate synthesis in excess [18].

Using GOT1 and GOT2 knockout and silencing strategies we found a preserved aspartate production by mitochondrial GOT2 showing that this part of the MAS was maintained in low glucose conditions. However, diminished NADH levels under low glucose conditions and the reversal of aspartate accumulation by exogenous lactate restoring NADH suggest that a diminished cytoplasmic MDH1 reaction (lower arm of the MAS) leads to the increase of *de novo* synthesized aspartate. GOT1 activity was rather increased, not suppressed, in low versus high glucose conditions. Thus, the diminished NADH-dependent MDH1 reaction in turn may lead to an accumulation of cytosolic OAA, thereby preventing further aspartate conversion to OAA by GOT1. As a limitation, rapid fractionation of cells for NADH and OAA measurements or ^2^H-labeling approaches to verify the glycolytic origin of NADH in high glucose conditions have not been performed in our study. Importantly, the reduced use in purine synthesis in the low glucose cells, which proliferated at slower rates, could contribute to aspartate accumulation and release. Whether aspartate use in protein synthesis is reduced under low glucose conditions is at present unknown and should be addressed in future studies.

As mentioned in the Introduction, a similar increase in *de novo* synthesis of aspartate from glutamine along with a collapse in NADH has been found recently in HCT-116 cancer cells treated with a GLUT inhibitor or by glucose removal. However, aspartate release to the medium was not investigated in that study [19]. Similar to our study, the authors found a reversal of aspartate accumulation by exogenous lactate and concluded that the diminished cytosolic NADH pool becomes rate-limiting for the MDH1 reaction, resulting in the accumulation of aspartate in the cytosol. In line with our study, GOT2 knockout reduced aspartate accumulation [19], suggesting that GOT2 was responsible for aspartate synthesis. While in our study, GOT1 silencing only enhanced aspartate in high glucose but not in low glucose conditions, the authors found GOT1 knockout to be effective also under glucose starvation [19]. The reason for this discrepancy is unknown, but a different level of GOT1 suppression might play a role. Still, both studies suggest that the disruption of the distal MAS is likely caused by an impaired MDH1 reaction due to a lack of NADH.

The glucose levels used in this study (0.2 mM compared to 10 mM) were chosen arbitrarily to mimic glucose deprivation in vivo. While average glucose levels in tumor interstitial fluid have been shown to be approximately half of plasma values [5], actual glucose levels in the extracellular environment have not been determined in different parts of a tumor with sufficient spatial resolution, to the best of our knowledge. Thus, the implications of our findings for adaptation of cancer cells to the tumor microenvironment in vivo are yet to be clarified. As another limitation, the glucose concentrations could be controlled only at the beginning of the experiment, and glucose can be consumed by the cancer cells rapidly [38]. In order to prevent prolonged severe glucose starvation, we replaced the medium in every experiment after 24 hours.

We found low-glucose enhanced aspartate release in both malignant and normal cells. Still, our and other studies show that components of the MAS are overexpressed in lung cancer and other cancers [53, 54]. Besides providing aspartate, the MAS, mediated by GOT1 and GOT2, also supports glycolysis by promoting the oxidation of NADH in the cytosol. Interestingly, proliferation defects caused by GOT2 knockout in pancreatic cancer cells have been shown to be rescued by exogenous supply with the electron acceptor pyruvate in vitro, but inhibiting pyruvate uptake did not modulate GOT2 sensitivity in vivo [44]. In contrast, a clear role of GOT2 in aspartate synthesis, rather than in MAS was found in a recent study investigating the effects of GOT1 and GOT2 deletions in hematopoietic stem cells in vivo [55]. Similar to our findings, GOT1 deficiency enhanced, while GOT2 deficiency reduced aspartate levels in that study, and GOT2, but not GOT1 deficiency, hampered stem cell function [55]. Thus, the main role of GOT2 and the MAS in cancers appears to be highly context-dependent [54]. In our study, GOT2 knockout did not affect proliferation at either glucose levels, neither in cells receiving aspartate from the medium (H23) nor in cells lacking exogenous aspartate (A549). Thus, GOT1 may compensate for the loss of GOT2. The results of our study, suggesting a low glucose-driven truncation of the MAS and release of aspartate, adds to the complexity. However, further studies are required to dissect the different roles of the MAS and extracellular aspartate in tumors and other tissues exhibiting metabolic stress conditions.

## Electronic supplementary material

Below is the link to the electronic supplementary material.

Supplementary Material 1

## Data Availability

Metabolomics data will be deposited at Zenodo (https://zenodo.org/records/18235195) upon publication.
