# Supplementary material for: A shift in the cellular redox state redirects aspartate for export under glucose deprivation
Source: Cancer Metab. 2026 Mar 3;14:4. doi: 10.1186/s40170-026-00420-x (PMC12955209; doi:10.1186/s40170-026-00420-x)
Supplement: Supplementary file 1 — Supplementary Material 1 [file 40170_2026_420_MOESM1_ESM.pdf]

Figure S1

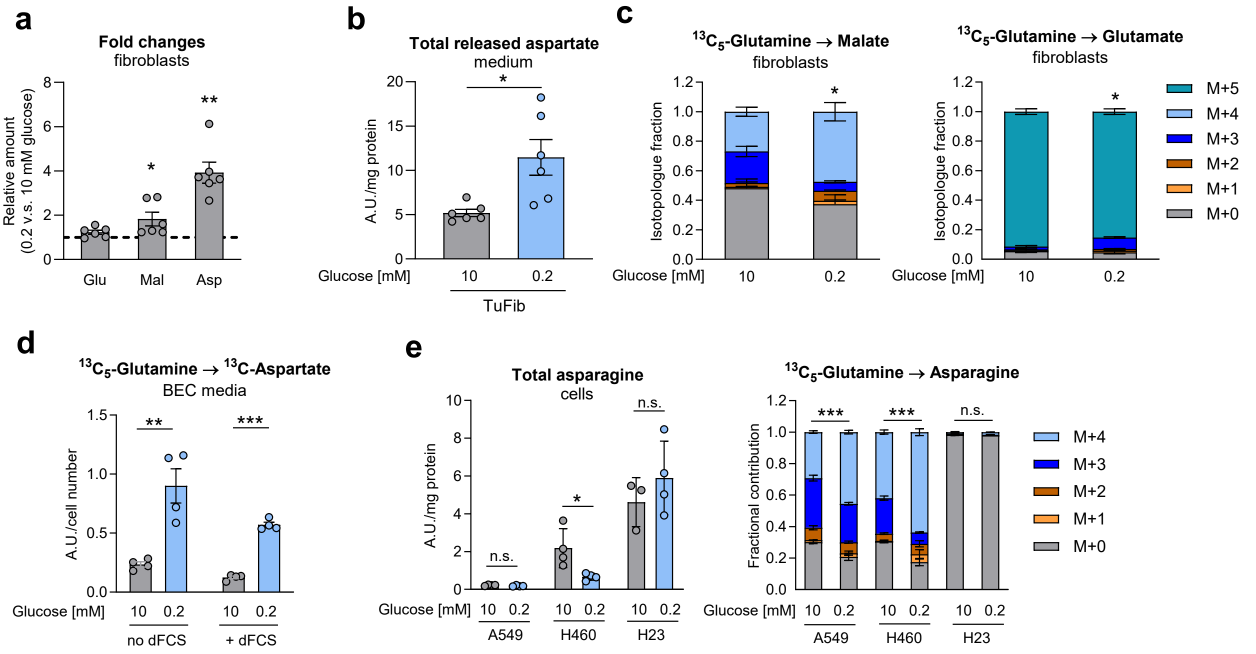

a
Fold changes
fibroblasts
Relative amount
(0.2 v.s. 10 mM glucose)
Glu
Mal
Asp
b
Total released aspartate
medium
A.U./mg protein
Glucose [mM]
10
0.2
TuFib
c
$^{13}C_5$-Glutamine → Malate
fibroblasts
Isotopologue fraction
$^{13}C_5$-Glutamine → Glutamate
fibroblasts
M+5
M+4
M+3
M+2
M+1
M+0
d
$^{13}C_5$-Glutamine → ^{13}C-Aspartate
BEC media
A.U./cell number
no dFCS
+ dFCS
e
Total asparagine
cells
A.U./mg protein
n.s.
A549
H460
H23
$^{13}C_5$-Glutamine → Asparagine
Fractional contribution

Figure S2

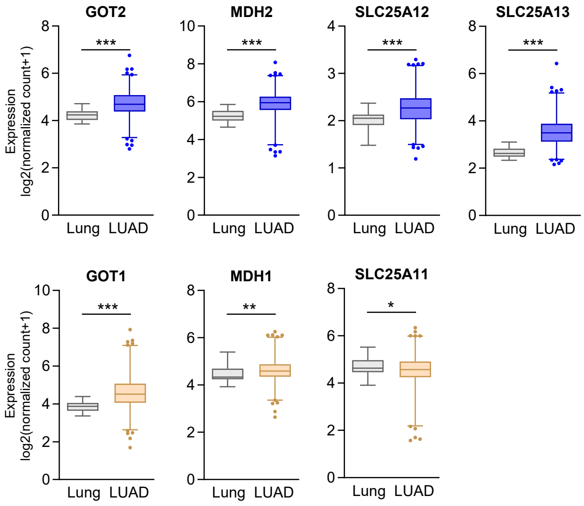
GOT2
MDH2
SLC25A12
SLC25A13
GOT1
MDH1
SLC25A11
Expression
log2(normalized count+1)
***
**
*
Lung
LUAD

Figure S3

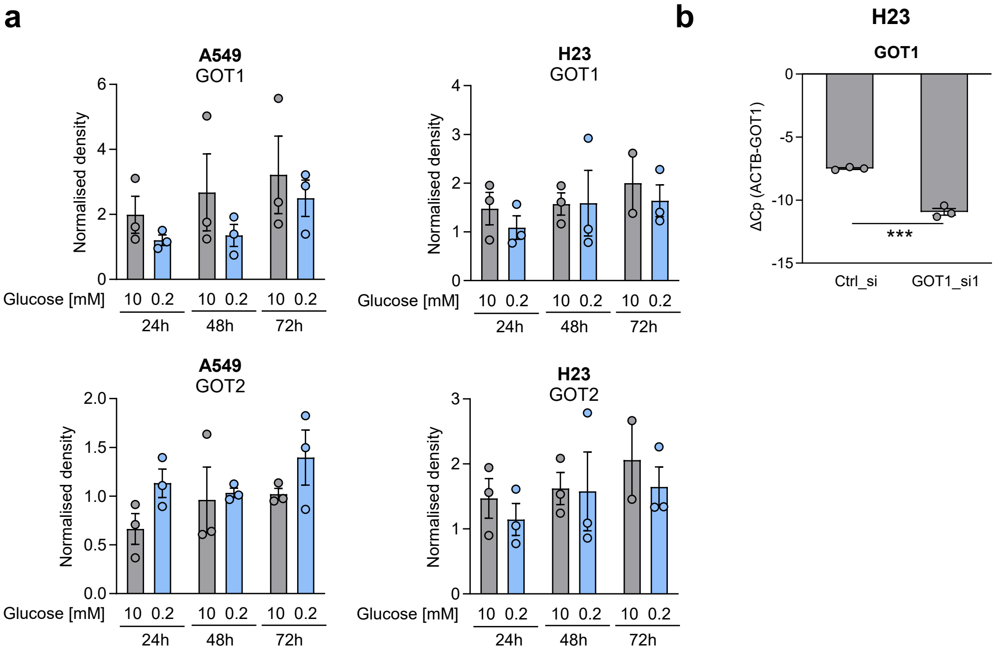

a
A549
GOT1
Normalised density
Glucose [mM]
10 0.2 10 0.2 10 0.2
24h 48h 72h
H23
GOT1
Normalised density
Glucose [mM]
10 0.2 10 0.2 10 0.2
24h 48h 72h
A549
GOT2
Normalised density
Glucose [mM]
10 0.2 10 0.2 10 0.2
24h 48h 72h
H23
GOT2
Normalised density
Glucose [mM]
10 0.2 10 0.2 10 0.2
24h 48h 72h
b
H23
GOT1
ΔCp (ACTB-GOT1)
***
Ctrl_si
GOT1_si1

Figure S4

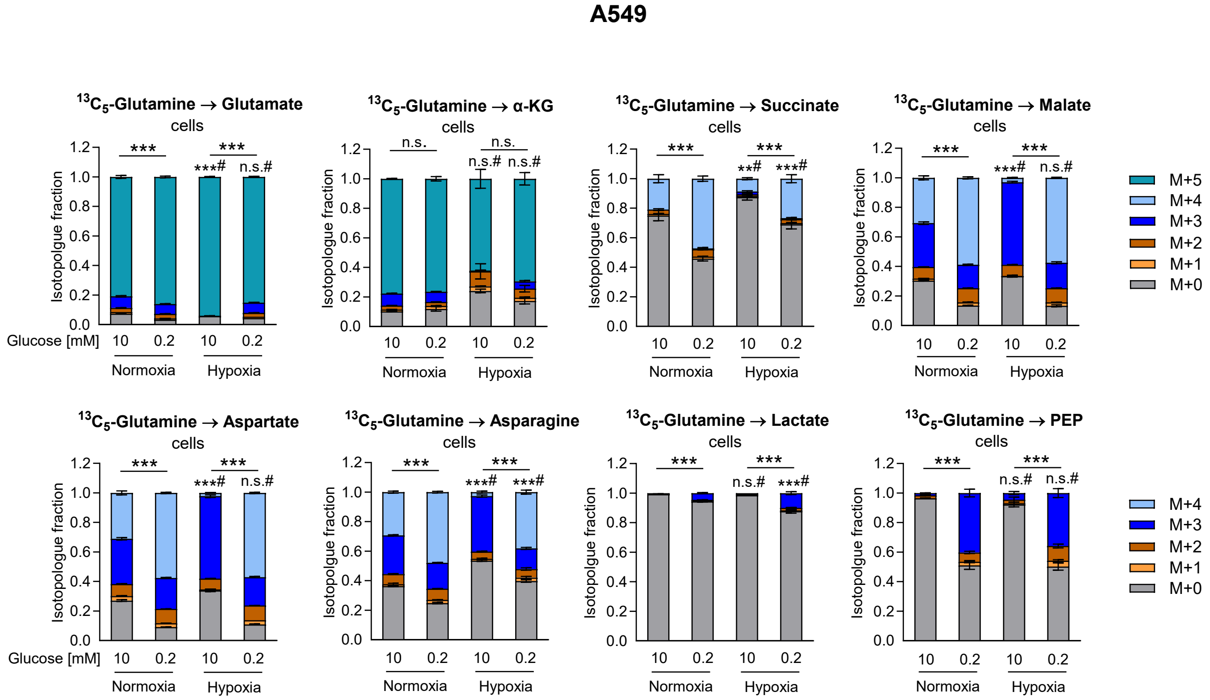
A549
$^{13}C_5$-Glutamine → Glutamate
cells
$^{13}C_5$-Glutamine → α-KG
cells
$^{13}C_5$-Glutamine → Succinate
cells
$^{13}C_5$-Glutamine → Malate
cells
$^{13}C_5$-Glutamine → Aspartate
cells
$^{13}C_5$-Glutamine → Asparagine
cells
$^{13}C_5$-Glutamine → Lactate
cells
$^{13}C_5$-Glutamine → PEP
cells
Isotopologue fraction
Glucose [mM]
10
0.2
Normoxia
Hypoxia
***
n.s.
***#
n.s.#
**#
M+5
M+4
M+3
M+2
M+1
M+0

Figure S5

H23

a

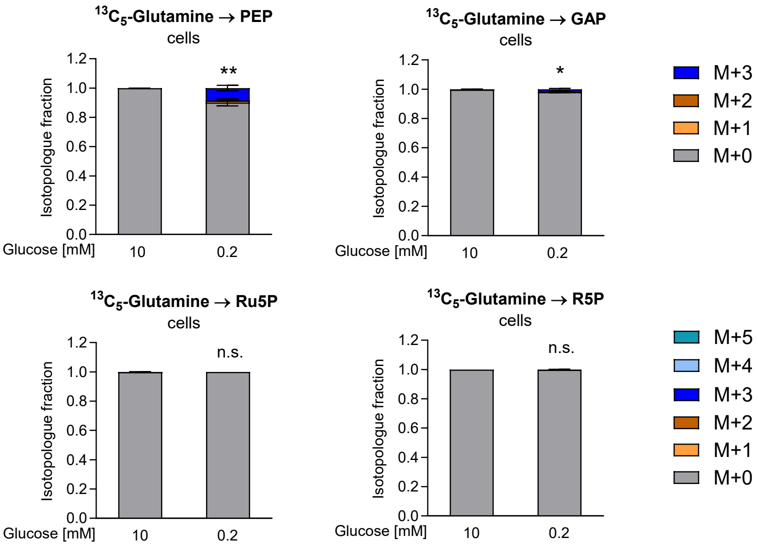

b

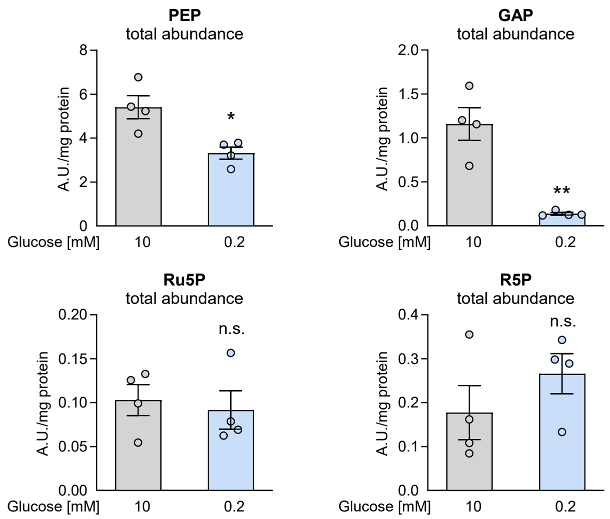

Figure S6

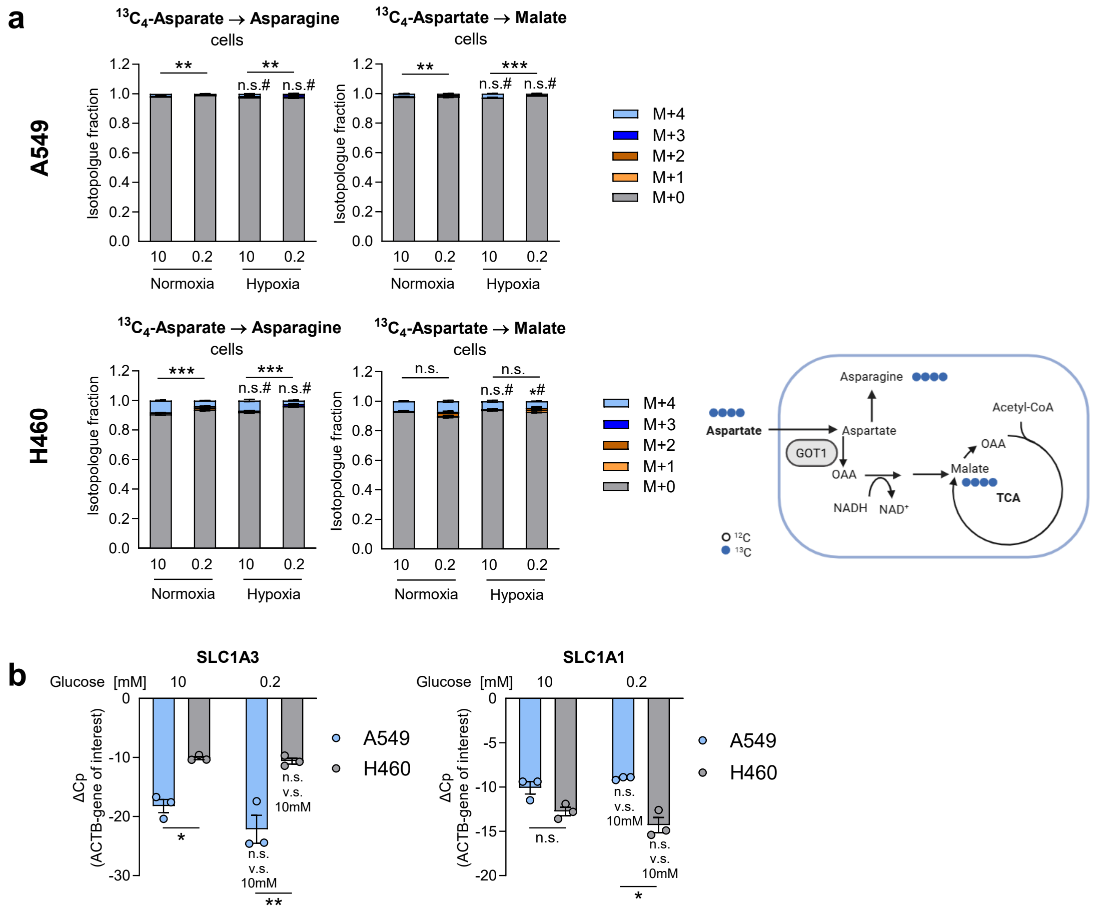

a
A549
H460
13C4-Asparate → Asparagine cells
13C4-Aspartate → Malate cells
Isotopologue fraction
Normoxia
Hypoxia
M+4
M+3
M+2
M+1
M+0
n.s.#
*#
n.s.
Asparagine
Aspartate
GOT1
OAA
NADH
NAD+
Malate
Acetyl-CoA
TCA
12C
13C
b
SLC1A3
SLC1A1
Glucose [mM]
ΔCp (ACTB-gene of interest)
A549
H460
n.s. v.s. 10mM

Figure S7

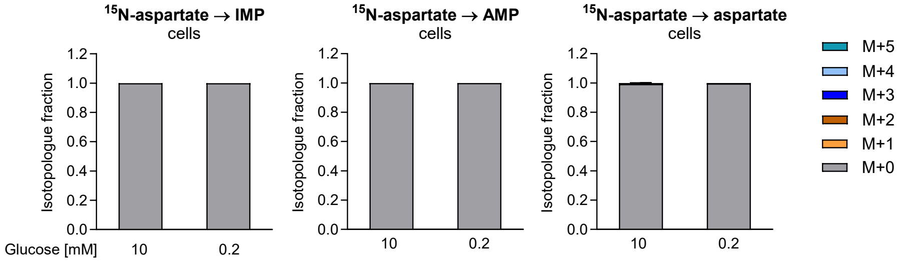

^{15}N-aspartate → IMP
cells
^{15}N-aspartate → AMP
cells
^{15}N-aspartate → aspartate
cells
Isotopologue fraction
1.2
1.0
0.8
0.6
0.4
0.2
0.0
Glucose [mM]
10
0.2
M+5
M+4
M+3
M+2
M+1
M+0

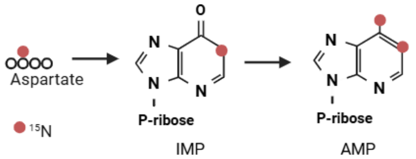

Aspartate
^{15}N
O
N
P-ribose
IMP
AMP

Supplementary Fig. legends, Konrad et al.

Fig. S1. Effects of low glucose availability on metabolism in different $^{13}C_5$-glutamine treated cells. (**a-d**) Uniformly labeled [$^{13}C_5$]-glutamine was administered to CAFs (**a-c**) or BEC (**d**) in media containing high (10 mM) or low (0.2 mM) glucose for 24 hours. (**a**) Fold changes of metabolites in CAFs and (**b**) release of total aspartate to the media supernatant. (**c**) Label enrichments in CAFs. (**d**) Release of ^{13}C-labeled aspartate to the media by BEC. (**e**) Asparagine labeling from $^{13}C_5$-glutamine in cancer cells and total abundance. Results are shown as mean +/- SEM from four (**d**,**e**) or six (**a-c**) independent experiments. Group comparisons were performed by one group Student's t-test v.s. no change (value = 1) (**a**) or Student's t-test (**b-e**). *$P < 0.05$; **$P < 0.01$; ***$P < 0.001$; n.s., not significant; (**c**,**e**) Statistical analysis was performed on relative enrichment of the highest isotopologue. Glu, glutamate; Mal, malate; Asp, aspartate.

Fig. S2. MAS components are overexpressed in lung adenocarcinoma compared to normal lung. Gene expression of enzymes and transporters mediating the initial steps of the MAS leading to aspartate synthesis (blue bars) or the subsequent steps (orange bars) in lung adenocarcinoma (LUAD, n=528) and non-involved lung (n=59) obtained from the publicly available TCGA dataset via the XENA database (https://xenabrowser.net/). Group comparisons were performed by Mann-Whitney U tests. * $P < 0.05$; ** $P < 0.01$; *** $P < 0.001$.

Fig. S3. GOT1 and GOT2 expression in cancer cells. (**a**) Treatment with different concentrations of glucose had no impact on GOT1 or GOT2 expression assessed by Western blot and subsequent quantification of relative intensities. Data were normalized to 0 hours and β-actin. (**b**) Silencing efficiency by GOT1_si1 analyzed by quantitative PCR. ACTB, β-actin. (**a**,**b**) Results are displayed as mean +/- SEM from three independent experiments. Group comparisons were performed by Student's t-test. (**a**) no significant differences were found on group comparisons. ***$P < 0.001$;

Fig. S4. Conversion of glutamine to TCA cycle intermediates, aspartate and PEP in hypoxia. A549 cells were treated with high or low glucose media in normoxia (ambient oxygen, 21%) or in hypoxia (0.5%) for 24 hours, followed by $^{13}C_5$-glutamine labeling for additional 24 hours at the respective conditions. Isotopologue fractions are shown as mean +/- SEM from four independent experiments. Group comparisons were performed using Two-way ANOVA with Tukey post-hoc analysis on relative enrichment of the highest isotopologue. $^{**}P < 0.01$; $^{***}P < 0.001$; n.s., not significant; $^{\#}$ versus normoxia.

Fig. S5. Partial gluconeogenesis in H23 cells. H23 cells were treated with the respective glucose levels for 24 hours and treated with the same media containing $^{13}C_5$-glutamine for additional 24 hours. Cells were analyzed by liquid chromatography - mass spectrometry. (**a**) Isotopologue fractions and (**b**) total abundance normalized to protein. Results are displayed as mean +/- SEM from four independent experiments. Statistical analysis was performed on the relative enrichment of M+3. Group comparisons were performed by Student's t-test. $^{*}P < 0.05$; $^{**}P < 0.01$; n.s., not significant; PEP, phosphoenolpyruvate; R5P, ribose-5-phosphate; Ru5P, ribulose-5-phosphate;

Fig. S6. Aspartate transporter expression and ^{13}C-aspartate catabolism in A549 and H460 cells. (**a**) Uniformly labeled [$^{13}C_4$]-aspartate was administered to A549 or H460 cells in media containing high (10 mM) or low (0.2 mM) glucose in ambient oxygen (normoxia) or 0.5% oxygen (hypoxia). Label enrichments in aspartate-derived metabolites. Right: Metabolic downstream pathways of aspartate. TCA cycle, tricarboxylic acid cycle; OAA, oxaloacetate; GOT1, cytoplasmic aspartate aminotransferase. Statistical analysis was performed on relative enrichment of the highest isotopologue. (**b**) qPCR for aspartate transporters in the different cell lines treated for 48 hours with high or low glucose conditions with medium replacement after 24 hours. Data were normalized to the expression of ACTB. (**a**,**b**) Results are displayed as mean +/- SEM from four (**a**) or three (**b**) independent experiments. (**a**,**b**) Group comparisons

were performed by Two-way ANOVA with Tukey post-hoc analysis. *$P < 0.05$; **$P < 0.01$; ***$P < 0.001$; n.s., not significant; # versus normoxia (**a**).

Fig. S7. Label enrichments in ^{15}N-aspartate treated cells. ^{15}N-aspartate was administered to A549 cells in media containing high (10 mM) or low (0.2 mM) glucose. Label enrichments in purine synthesis intermediates was assessed by liquid chromatography – mass spectrometry. No label transfer was found. Results are displayed as mean +/- SEM from four independent experiments. Right: Putative labeling patterns from ^{15}N-aspartate in inosine monophosphate (IMP) and adenosine monophosphate (AMP).
